# Immune hemolytic anemia associated with the use of immune checkpoint inhibitors: a scoping review

**DOI:** 10.3389/fimmu.2025.1586426

**Published:** 2025-07-03

**Authors:** Juan-Manuel Hernandez-Martinez, Eduardo Rios-Garcia, Cittim B. Palomares-Palomares, Oscar Arrieta

**Affiliations:** ^1^ Thoracic Oncology Unit and Experimental Oncology Laboratory, Instituto Nacional de Cancerologiía de Meíxico (INCan), Mexico City, Mexico; ^2^ Secretaría de Ciencia, Humanidades, Tecnología e Innovación (SECIHTI)-Instituto Nacional de Cancerología, Mexico City, Mexico

**Keywords:** immune-related adverse events, autoimmune hemolytic anemia, immune checkpoint inhibitors, drug-induced immune hemolytic anemia, risk - benefit

## Abstract

**Background:**

Immune-hemolytic anemia (IHA) is a rare immune-related adverse event (irAE) in cancer patients treated with immune-checkpoint inhibitors (ICIs). Although several cases of ICI-associated IHA have been reported, few attempts have been made to collate available information. This scoping review aims to provide a comprehensive description of the clinical features of ICI-associated IHA.

**Methods:**

PubMed and Web of Science Core Collection databases were searched for articles published in English from January 2006 to January 2025 on ICI-associated IHA. Only full-text publications reporting the clinical characteristics of patients with ICI-associated IHA were included. Two authors independently assessed the search results for eligibility and extracted the following information: author, publication year, patient characteristics, and IHA features.

**Results:**

Among 54 publications, published between July 2014 and March 2024, 92 cases of ICI-associated IHA were identified, revealing a high proportion of cases in patients with melanoma (45.2%) and non-small-cell lung cancer (31%). Approximately half of the cases occurred in patients receiving ICIs as first-line systemic therapy, with IHA manifesting after a median of 3 cycles. The most frequent triggering ICIs were pembrolizumab (41.3%) and nivolumab (26.1%). A high proportion of cases involved patients with a medical history of hematolymphoid tumors (34.8%), hypertension (15.2%), and anemia/AIHA (15.2%). Initial management involved ICI discontinuation (94.6%), high-dose glucocorticoids therapy (97.8%), and transfusion support (63%), with treatment responses achieved in most cases (91.3%). Only 2 cases reported fatal outcomes. IHA relapse was documented in only 7 of 23 (30.4%) patients who were rechallenged with an ICI.

**Conclusions:**

To the best of our knowledge, this is the largest scoping review of population-based studies, case reports, and case series on ICI-associated IHA. The evidence reviewed suggests that patients with specific comorbidities may be at higher risk of developing ICI-associated IHA. In the absence of predictive tools to individually estimate the risk of this complication, a list of frequently reported co-occurring conditions in cases of ICI-associated IHA may help select patients who could benefit from closer surveillance.

## 1 Background

Immune checkpoint inhibitors (ICIs), monoclonal antibodies (mAbs) that interrupt inhibitory signaling pathways in T-cells by binding to co-inhibitory receptors or their ligands, have revolutionized cancer treatment. Specifically, the use of ICIs that block *cytotoxic T-lymphocyte-associated antigen 4* (CTLA-4), *programmed cell death 1* (PD-1) protein, and *programmed cell death 1 ligand 1* (PD-L1) has resulted in unprecedented durable responses and long-term survival rates in cancer patients with different tumor types (1).

As of May 2025, ICIs approved by the U.S. Food and Drug Administration (FDA) and the European Medicines Agency (EMA) include CTLA-4 inhibitors (ipilimumab and tremelimumab), PD-1 inhibitors (pembrolizumab, nivolumab, cemiplimab, dostarlimab, retifanlimab, toripalimab, tislelizumab), PD-L1 inhibitors (atezolizumab, durvalumab, avelumab, cosibelimab), and a lymphocyte activation gene-3 (LAG-3) inhibitor (relatlimab) in combination with nivolumab (2).

Several studies have shown that ICIs have a better safety profile than other treatment modalities including cytotoxic chemotherapy and radiotherapy. Despite the relative safety of ICIs, their use has been associated with a distinct class of adverse events known as immune-related adverse events (irAEs), which arise as an unintended consequence of an overactivated immune system. Clinical manifestations and presentation are diverse and may involve virtually any organ or system of the body. Common irAEs that have been extensively documented and are relatively well characterized include cutaneous (pruritus and rash), endocrine (hyperthyroidism and hypothyroidism), gastrointestinal (diarrhea and colitis), and pulmonary (pneumonitis) manifestations (3).

Given the rapidly expanding indications for ICIs, the number of patients presenting relatively rare irAEs is increasing. Thus, it is important for oncologists to be aware of other less documented irAEs that arise in patients treated with ICIs, including hematological irAEs (hem-irAEs). Although hem-irAEs are generally uncommon, neutropenia and different types of thrombocytopenia and anemia are among the most frequent hem-irAEs (4–6). Furthermore, cases of acquired hemophilia A, hemophagocytic lymphohistiocytosis (HLH), and autoimmune cytopenia (AICs), including pancytopenia, neutropenia, thrombocytopenia, immune thrombocytopenic purpura (ITP), thrombotic thrombocytopenic purpura (TTP), pure red cell aplasia (PRCA), anemia, aplastic anemia, hemolytic anemia (HA), and autoimmune hemolytic anemia (AIHA), have all been described in patients treated with ICIs (7–15).

AIHA is a subset of hemolytic anemia in which immunoglobulin (Ig)G, IgM, or IgA autoantibodies cause the premature destruction of red blood cells (RBCs). Depending on the temperature at which autoantibodies display maximal antigen binding, AIHA cases can be classified as warm-antibody AIHA (wAIHA) or cold-antibody AIHA (cAIHA). wAIHA is caused by polyclonal autoantibodies, usually IgG, that optimally bind to RBC antigens (Rh proteins or glycophorins A-D) at 37°C. Antigen-dependent IgG oligomerization, complement binding and complement activation is generally low. Thus, IgG-coated RBCs are primarily removed by reticuloendothelial macrophages outside of the circulation (extravascular hemolysis) (16, 17). Evans syndrome (ES) is a rare autoimmune disorder in which wAIHA occurs simultaneously or sequentially with thrombocytopenia.

Cold antibodies cause two clinical conditions, cold agglutinin disease (CAD) and paroxysmal cold hemoglobinuria (PCH), which are defined by the isotype of the causal cold-reacting autoantibody. CAD is mediated by IgM autoantibodies that bind to the “I” antigen of RBCs with maximal reactivity at 4°C, causing RBC agglutination and complement activation. Hemolysis may be extravascular or intravascular when complement activation is complete. In contrast, PCH is caused by IgG autoantibodies directed against the “P” antigen of RBCs. These antibodies, called Donath-Landsteiner antibodies, are biphasic hemolysins that bind to RBCs and fix complement at cold temperatures; however, they detach from RBCs and activate complement as the temperature rises to 37°C (18). Patients whose serological results meet the criteria for both wAIHA and cAIHA are diagnosed with “mixed AIHA”. AIHA driven by IgA or warm-IgM are considered atypical forms that remain a diagnostic and therapeutic challenge.

AIHA is considered primary, also known as ‘idiopathic’ (i.e., of unknown cause), in the absence of an underlying disorder and secondary when one is present (16, 19). Immune hemolysis that accompanies or follows the use of certain drugs is commonly referred to as ‘drug-induced immune hemolytic anemia’ (DIIHA) and is classified by different authors as a secondary form of AIHA, a distinct AIHA subgroup, or as a distinct category of secondary immune hemolysis (16, 20, 21). Throughout, the term ‘ICI-associated immune hemolytic anemia’ (ICI-associated IHA) will be used to avoid implying causation when referring to cases of immune hemolytic anemia secondary to ICI therapy. It has been estimated that DIIHA accounts for 10% of all AIHA cases (22). A study that used the database of the FDA to investigate the frequency of DIIHA secondary to ICI therapy estimated that the absolute frequency of this complication could be as high as 0.25%, more frequently caused by anti-PD-(L)1 agents than by anti-CTLA-4 therapy (23).

Although several cases of ICI-associated IHA have been published, and a substantial number of reviews on this complication can be found in the literature, few attempts have been made to collate available information and provide a complete picture of this irAE (24).

## 2 Methods

### 2.1 Criteria for considering studies for this review

#### 2.2.1 Types of studies included

Only full-text publications reporting the clinical characteristics of patients with ICI-associated IHA were included. ICI-associated IHA was defined according to the following criteria proposed by Leaf et al. (25): (1) an abrupt decrease in Hb ≥2 g/dL; (2) at least two laboratory findings of hemolysis (elevated lactate dehydrogenase [LDH], elevated indirect bilirubin, elevated reticulocyte count/percentage, low serum haptoglobin, positive serum free hemoglobin, positive urine hemosiderin, or presence of spherocytes on peripheral blood smear); (3) AIHA occurrence after ICI initiation; and (4) exclusion of other causes of anemia. There were no restrictions on the type of publication and all original case reports, case series, and population-based studies were included. Articles written in languages other than English were excluded from this review.

### 2.2 Search methods for identification of studies

#### 2.2.1 Electronic search

Two databases (PubMed and Web of Science core collection) were queried for articles published from January 2006 to January 2025 using the following terms: (“autoimmune hemolytic anemia” OR “autoimmune haemolytic anaemia” OR “immune hemolytic anemia” OR “immune haemolytic anaemia” OR “immune-induced haemolysis” OR “immune-induced hemolysis”OR “drug-induced immune hemolytic anemia” OR “drug-induced immune haemolytic anaemia” OR “hemolytic anemia” OR “haemolytic anaemia” OR “AIHA” OR “DIIHA” OR “cold agglutinin disease” OR “cold agglutinin syndrome” OR “cold agglutinin” OR “paroxysmal cold hemoglobinuria” OR “paroxysmal cold haemoglobinuria”) AND (“immune checkpoint inhibitor” OR “immune checkpoint blockade” OR “atezolizumab” OR “pembrolizumab” OR “nivolumab” OR “durvalumab” OR “avelumab” OR “ipilimumab” OR “tremelimumab” OR “anti-ctla-4” OR “anti-pd-1” OR “anti-pd-l1” OR “anti-PD1” OR “anti-PDL1” OR “anti-ctla4” OR “PD-1” OR “PD1” OR “PD-L1” OR “CTLA-4”). The search was last updated on the 15^th^ of January 2025.

#### 2.2.2 Searching other sources

The references of the included studies were manually searched to identify additional relevant studies. Additionally, experts on the topic were consulted for further relevant literature.

### 2.3 Data collection and analysis

#### 2.3.1 Selection of studies

Two authors assessed the eligibility of the articles retrieved from the search strategy by screening titles and abstracts. Full-text publications were retrieved from potentially eligible articles. Two authors independently evaluated whether full-text publications met the inclusion criteria by critically reading the entire article. Discrepancies were resolved with the input of a third author. Assessors were not blinded to the author, institution, or journal of publication, as the review authors were familiar with most studies and the typographical layout of the journals.

#### 2.3.2 Data extraction and management

Two authors independently extracted data using data extraction forms specifically designed for this review, with discrepancies resolved by a third assessor. The extracted data included author, publication year, patient characteristics (age, sex, tumor type, comorbidities, and medical history), and features of ICI-associated IHA (diagnostic term, diagnostic workup, type and number of previous systemic therapies, triggering ICI, number of cycles at onset, time to onset, treatment, treatment response, number of fatal outcomes, ICI rechallenge, and relapse after rechallenge). No assumptions were made during data collection. Only explicit statements regarding the clinical characteristics of patients and the features of IHA were used in the analysis.

#### 2.3.3 Data analysis

The information extracted was summarized using counts and valid percentages (non-missing values) for nominal variables and medians (with ranges) for ordinal and quantitative variables with asymmetric distributions. Findings are reported descriptively. All calculations were performed using SPSS statistical software version 29 (IBM Corp). The PRISMA flow diagram was created in R using the PRISMA2020 package and Shiny App, as previously described (26).

## 3 Results

**Figure 1:**
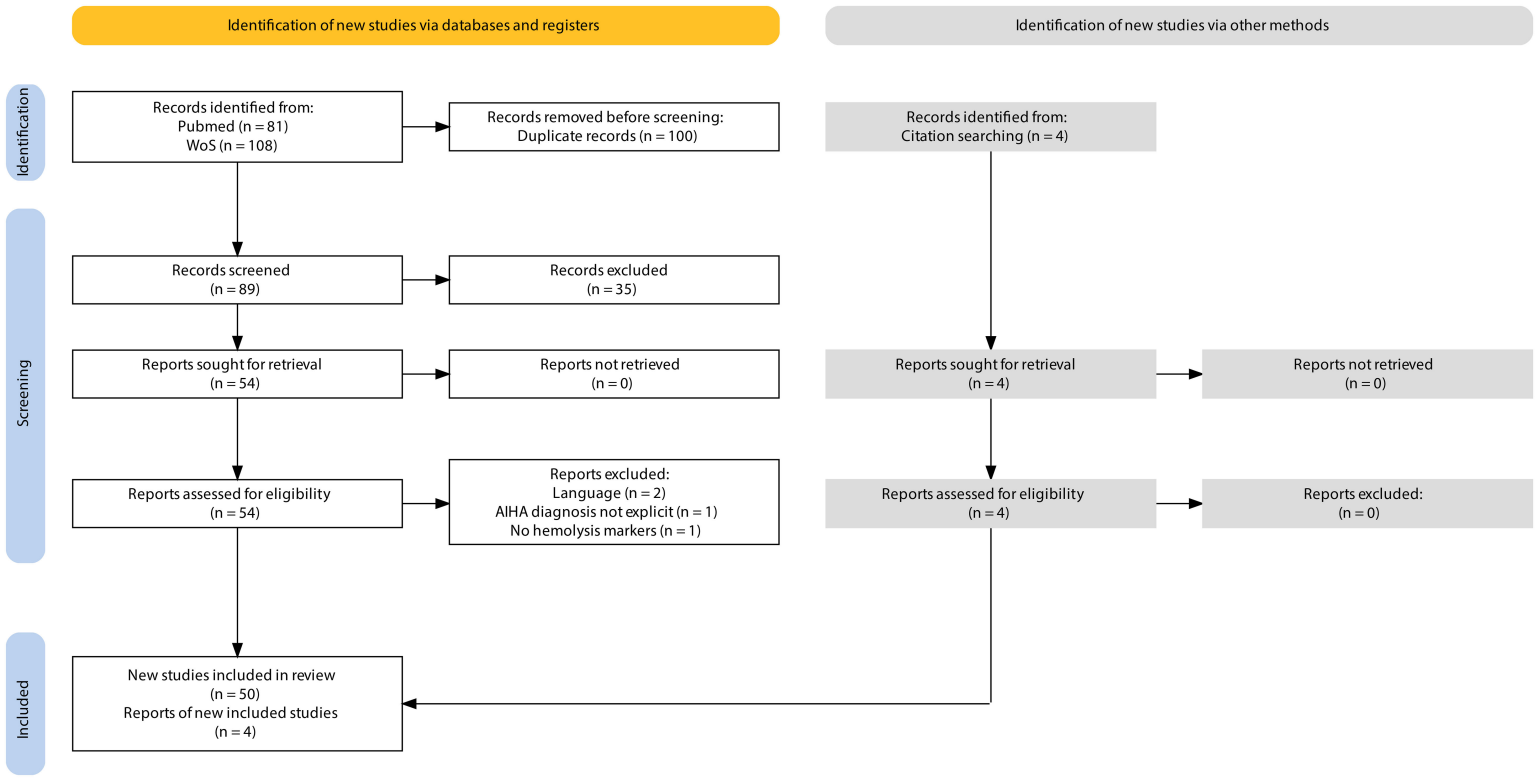
PRISMA. Flow diagram for systematic reviews.

The search yielded a total of 189 results. We excluded 100 results as they were duplicates. This resulted in 89 publications with available titles and abstracts. After excluding 35 publications based on title and abstract screening, 54 full-text publications were assessed for potential inclusion by critically reviewing the entire article. A further 4 records were excluded as they did not meet the inclusion criteria. Four additional records that met the inclusion criteria were identified from the references of the included studies (**Figure 1**). This review ultimately included 54 articles on ICI-associated IHA (44 case reports and 10 studies with patient-level information), published between July 2014 and March 2024. The information extracted from these publications, documenting 92 cases of ICI-associated IHA, is presented in **Supplementary Table 1**.

The clinicopathological characteristics and features of ICI-associated IHA in these patients are summarized in **Table 1**. The median age at cancer diagnosis was 67 years (range: 18–89 years), with most reported cases occurring in males (55 of 91 [60.4%]). Among the 84 cases in which the primary tumor type was documented, the most frequently reported malignancies were melanoma (38 [45.2%] patients), NSCLC (26 [31%] patients), urothelial carcinoma, small-cell lung cancer, renal cell carcinoma, and Hodgkin lymphoma (each in 3 [3.7%] patients), followed by breast cancer (2 [2.5%] patients). The remaining cases correspond to six patients diagnosed with acute myeloid leukemia, esophageal adenocarcinoma, cutaneous small-cell cancer, pancreatic adenocarcinoma, colorectal cancer, and hepatocellular carcinoma (each in one [1.2%] patient). Of the 64 cases documenting the number of lines of systemic therapy at IHA onset, the majority (34 [50.7%]) occurred in the first-line, with the remaining cases distributed across the 2^nd^ (14 [20.9%] cases), 3^rd^ (13 [19.4%] cases), and ≥4^th^ (6 [9%]) lines. The median time from ICI initiation to IHA onset was approximately 62 days (range: 6–780 days), corresponding to a median of three cycles (range: 1–39) of ICI therapy. The most frequently reported triggering ICIs were pembrolizumab (38 of 92 [41.3%] cases) and nivolumab (24 of 92 [26.1%] cases), followed by nivolumab + ipilimumab (11 of 92 [12%] cases), atezolizumab (10 of 92 [10.9%] cases), and ipilimumab (6 of 92 [6.5%] cases). Finally, ipilimumab + pembrolizumab, penpulimab (anti-PD-1), and an unspecified anti-PD-1 agent were associated with one case each (1.1%). No cases involving durvalumab, avelumab, or tremelimumab were identified. The most frequently used diagnostic terms were AIHA (in 69 [75%] cases), wAIHA (11 [12%] cases), Evans syndrome (4 [4.3%]), CAD (3 [3.3%]), CAS (2 [2.2%]), secondary CAD (2 [2.2%]), and cAIHA (1 [1.1%]), with no cases identified explicitly reporting the occurrence of mixed AIHA or PCH. Among the 88 cases in which a DAT was performed as part of the diagnostic workup, 72 (81.8%) reported a positive result, with most cases reporting the isotype of the autoantibodies identified (52 of 72 [72.2%]).

**Table 1:** Clinicopathological characteristics of patients with ICI-associated IHA identified in the literature.

Characteristic	Total
	N = 92
	n (%)
Age at onset	
Median (range)	67 (18-89)
Sex	
Not reported	1 (1.1)
Reported	91 (98.9)
Male	55 (60.4)
Female	36 (39.6)
Primary tumor type	
Not reported	8 (8.7)
Reported	84 (91.3)
Melanoma	38 (45.2)
Non-small-cell lung cancer	26 (31)
Urothelial carcinoma	3 (3.6)
Small-cell lung cancer	3 (3.6)
Renal cell carcinoma	3 (3.6)
Hodgkin lymphoma	3 (3.6)
Breast Cancer	2 (2.4)
Acute myeloid leukemia	1 (1.2)
Esophageal adenocarcinoma	1 (1.2)
Cutaneous small-cell cancer	1 (1.2)
Pancreatic Adenocarcinoma	1 (1.2)
Colorectal	1 (1.2)
Hepatocellular carcinoma	1 (1.2)
Treatment line at onset	
Not reported	25 (27.2)
Reported	64 (72.8)
1^st^ line	34 (50.7)
2^nd^ line	14 (20.9)
3^rd^ line	13 (19.4)
≥ 4^th^ line	6 (9.0)
Time to onset	
Cycles (Median [range])	3 (1-39)
Days (Median [range])	62 (6-780)
Triggering ICI	
Pembrolizumab (anti-PD-1)	38 (41.3)
Nivolumab (anti-PD-1)	24 (26.1)
Nivolumab + Ipilimumab	11 (12)
Atezolizumab (anti-PD-L1)	10 (10.9)
Ipilimumab (anti-CTLA-4)	6 (6.5)
Ipilimumab + Pembrolizumab	1 (1.1)
Penpulimab (anti-PD-1)	1 (1.1)
Unspecified Anti-PD-1	1 (1.1)
Diagnosis	
AIHA unspecified	69 (75)
wAIHA	11 (12)
CAD	3 (3.3)
Secondary CAD	2 (2.2)
CAS	2 (2.2)
cAIHA	1 (1.1)
PCH	0 (0)
Mixed AIHA	0 (0)
Evans syndrome	4 (4.3)
DAT at diagnostic workup	
Not reported	2 (2.2)
Not performed	2 (2.2)
Performed	88 (95.7)
Negative result	16 (18.2)
Positive result	72 (81.8)
Isotype not reported	20 (27.8)
Isotype reported	52 (72.2)
IgG+ C3–	20 (38.5)
IgG+ C3+	17 (32.7)
IgG– C3+	15 (28.8)

Percentages were calculated using non-missing values. Percentages may not sum to 100 due to rounding.

AIHA, autoimmune hemolytic anemia; CAD, cold agglutinin disease; cAIHA, cold AIHA; CAS, cold agglutinin syndrome; DAT, direct antiglobulin test; ICI, immune checkpoint inhibitor; PCH, Paroxysmal cold hemoglobinuria; US GC, unspecified glucocorticoid.

Reported management strategies, treatments, and clinical outcomes are summarized in **Table 2**. Initial management of ICI-associated IHA involved the suspension of ICI therapy in 87 (94.6%) patients, transfusion support in 58 (63%) patients, and first-line pharmacological therapy in 90 (97.8%) patients, predominantly high-dose glucocorticoid therapy with prednisone (33 [36.7%]), methylprednisolone (17 [18.9%]), and prednisolone (13 [14.4%]). Only 10 cases (11.1%) reported the use of rituximab combined with glucocorticoids as first-line treatment. Treatment responses were achieved in 84 (91.3%) cases, including complete responses in 43 (46.7%) patients. Only two (2.2%) cases reported a fatal outcome. Twenty-three of 87 (25%) patients in whom ICI therapy was initially withheld resumed ICI therapy. Most patients who were rechallenged with an ICI did not develop any irAE (14 of 23 [60.9%]). However, recurrence of ICI-associated IHA was the most frequent irAE after rechallenge (7 of 9 [77.8%]). Two patients who were rechallenged developed an irAE other than IHA. In contrast, the majority of patients with ICI-associated IHA who continued on ICI therapy developed an irAE (4 of 5 [80%]), predominantly IHA (3 of 4 [75%]).

**Table 2:** Management and outcomes of patients with ICI-associated IHA.

Variable	Total
	N = 92
	n (%)
ICI withheld	
No	5 (5.4)
Yes	87 (94.6)
Transfusion support	
No	34 (37)
Yes	58 (63)
1^st^ line treatment for ICI-associated IHA	
Drug treatment	90 (97.8)
Prednisone	33 (36.7)
MPL	17 (18.9)
Prednisolone	13 (14.4)
Unspecified glucocorticoid	10 (11.1)
Rituximab + Prednisone	4 (4.4)
Rituximab + US GC	4 (4.4)
MPL + Prednisone	4 (4.4)
Dexamethasone	3 (3.3)
Rituximab + MPL	2 (2.2)
Treatment response	
No response	8 (8.7)
Response	41 (44.6)
Complete response	43 (46.7)
Fatal IHA	
No	90 (97.8)
Yes	2 (2.2)
ICI rechallenge	
N/A*	5
No	64 (73.6)
Yes	23 (26.4)
irAE after rechallenge	
No	14 (60.9)
Yes	9 (39.1)
IHA	7 (77.8)
Rash	1 (1.1)
Pneumonitis	1 (1.1)
irAE in patients who continued on ICI therapy	
No	1 (20)
Yes	4 (80)
IHA	3 (75)
Other	1 (25)

Percentages were calculated using non-missing values. Percentages may not sum to 100 due to rounding. The numbers within a column may not add to the total number reported for “Treatment” because patients who received transfusion support and drug treatment were counted for each intervention but only once for “Treatment”.

*Patients in whom ICI therapy was not withheld are shown but they were not included in the rechallenge analysis.

ICI, immune checkpoint inhibitor; IHA, immune hemolytic anemia; irAE, immune-related adverse event; MPL, methylprednisolone; N/A, not applicable; US GC, unspecified glucocorticoid.

**Table 3** presents frequently reported components of the health history of patients with ICI-associated IHA. The presence or absence of comorbid conditions was documented in 56 of 92 (60.9%) cases; 46 cases occurred in patients with comorbidities. Specifically, there was a high proportion of cases in patients with a past medical history of hematolymphoid tumors (16 of 46 [34.8%] cases), hypertension (7 of 46 [15.2%] cases), anemia/AIHA (7 of 46 [15.2%] cases), diabetes (5 of 46 [10.8%] cases), and solid tumors (4 of 46 [8.7%]). We identified several cases of patients presenting with other irAEs (31 of 52 [59.6%]), receiving concomitant medications (26 of 37 [70.3%]), with a history of smoking (5 of 8 [62.5%]), and previous blood transfusion (4 of 5 [80%]).

**Table 3:** Clinical information reported in cases of ICI-associated IHA.

Characteristic	Total
	N=92
	n (%)
Comorbidities	
Not reported	36 (39.1)
Reported	56 (60.9)
Comorbidities absent	10 (17.9)
Comorbidities present	46 (82.1)
Hematolymphoid tumors	16 (34.8)
CLL	12 (75)
MZL	4 (25)
Hypertension	7 (15.2)
Diabetes	5 (10.8)
Hypothyroidism	5 (10.8)
Multiple primary solid tumors	4 (8.7)
Prostate Cancer	2 (50)
Breast Cancer	2 (50)
AIHA	4 (8.7)
Anemia	3 (6.5)
Other comorbidities	29 (74.4)
Concurrent irAEs	
Not reported	40 (43.5)
Reported	52 (56.5)
Yes	31 (59.6)
No	21 (40.4)
Concomitant medication use	
Not reported	55 (59.8)
Reported	37 (40.2)
Yes	26 (70.3)
No	11 (29.7)
Smoking status	
Not reported	84 (91.3)
Reported	8 (8.7)
Positive	5 (62.5)
Negative	3 (37.5)
Previous transfusions	
Not reported	87 (94.6)
Reported	5 (5.4)
Yes	4 (80)
No	1 (20)

Percentages were calculated using non-missing values. Percentages may not sum to 100 due to rounding. The numbers within a column may not add to the total number reported for “Comorbidities present” because patients who had more than one comorbid condition were counted for each condition but only once for “Comorbidities present” and because only comorbid conditions that were reported in at least 1% of cases are shown.

AIHA, autoimmune hemolytic anemia; CLL, chronic lymphocytic leukemia; irAEs, immune-related adverse events; MZL, marginal zone lymphoma.

## 4 Discussion

This review aimed to quantitatively describe and summarize the available information on ICI-associated IHA to provide a comprehensive picture of this intriguing complication. Given the predominance of case reports and case series, which often constitute the primary sources of information for rare adverse events, a scoping review methodology was deemed the most appropriate approach to address the complexity of ICI-associated IHA. In reviewing the scientific literature, several factors that complicate the identification, management, and study of ICI-associated IHA were identified, including significant variability in terminology, diagnostic criteria, and reporting standards.

In line with a previous systematic review (20), we found considerable variation in the terminology used for the diagnosis of ICI-associated IHA. Cases of ICI-associated IHA were referred to by many names that denote their presumed causality and underlying mechanism (e.g., drug-associated/induced/mediated AIHA, antibody-mediated hemolytic anemia, immune-mediated hemolytic anemia, and immune hemolytic anemia). The limited data on the pathophysiology of DIIHA, which remains largely understudied in patients with cancer treated with ICIs, may partly explain the diverse nomenclature used in reports of ICI-associated IHA. Notably, only two reports of ICI-associated IHA used the term cold agglutinin syndrome (CAS) to refer to cases of clinically significant cold agglutinins secondary to ICI therapy or other conditions. In two other reports, patients were diagnosed with AIHA despite testing positive for cold agglutinins. Furthermore, important diagnostic information was often omitted or inadequately reported in cases of ICI-associated IHA. For instance, the reference ranges for laboratory tests were frequently missing, which poses a problem for borderline values since reference intervals may vary between different populations, laboratories, and analytical methods. Similarly, cold agglutinin titers and thresholds used to establish positivity were not reported in three cases.

Despite important efforts to harmonize definitions and diagnostic criteria (16), the terminology used to describe AIHA, particularly cAIHA, remains unclear. As previously mentioned, AIHA and its subtypes have traditionally been classified as primary or secondary. However, cold agglutinin disease (CAD), the primary form of cAIHA, is currently recognized as a distinct clinicopathological entity mediated by cold agglutinins and driven by an underlying clonal B-cell lymphoid proliferation that does not fulfill the criteria for B-cell lymphoma (27, 28). Given the significant histological, immunophenotypic, and molecular overlap between CAD and MZL, distinguishing primary from secondary cases can be particularly difficult (28). An excellent example of this was provided by Karki et al. who determined that the most likely etiology of ‘secondary CAD’ in a patient with lung cancer treated with pembrolizumab was a low-grade B-cell lymphoma with lymphoplasmacytic differentiation negative for the *MYD88* mutation (42).

This review also found considerable variation between reports in the primary and ancillary investigation of ICI-associated IHA. Although this variation may be explained by the diversity of the clinical scenarios described in these reports, it raises questions regarding the extent of laboratory testing in patients suspected of having ICI-associated IHA, particularly regarding the exclusion of alternative causes. Existing guidelines are not entirely clear on the extent to which alternative causes should be investigated (19, 22, 29, 30).

British guidelines on primary AIHA indicate that AIHA can be diagnosed when there is laboratory evidence of hemolysis, a positive DAT result, and clinical evaluation has excluded alternative causes such as DIIHA, delayed hemolytic transfusion reactions, and post-transplantation alloimmune hemolysis (19). According to these guidelines, the next step after AIHA has been diagnosed is to investigate for an underlying associated condition and to determine the subtype of AIHA (19). The above recommendations seem to suggest that further investigation is not required once DIIHA has been established, which appears to be an uncommon strategy among cases of ICI-associated IHA, while raising questions regarding which criteria can be used to reliably establish DIIHA as a final diagnosis.

Guidelines for the management of secondary AIHA and DIIHA indicate that further serological investigation is required only in cases in which there is clear evidence of hemolysis and a good temporal relationship with the suspected drug, unless the suspected drug is known to act via drug-independent antibodies (22). Others have interpreted this to mean that further studies are not indicated if there is an adequate temporal relationship between the administration of the drug and the onset of symptoms or if the suspected drug is associated with drug-independent hemolysis (31).

However, difficulties arise when implementing these guidelines in patients with DIIHA secondary to ICI therapy. First, this review found a high proportion of DAT negative results (17.5%) in patients with ICI-associated IHA, with an important contribution to this estimate from the study by Leaf et al., in which 38% of patients had negative DAT results (25). Furthermore, the interpretation of the DAT can be complex because it can be positive in up to 0.1% of healthy blood donors and 8% of hospitalized patients without evidence of hemolysis, or negative in up to 10% of patients with clear evidence of AIHA (16). Thus, DAT results are insufficient for the diagnosis of AIHA or its rejection. In the absence of a definitive diagnostic test, DAT-negative AIHA is a difficult diagnosis of exclusion, partly supported by response to corticosteroid therapy.

Although clinical evaluation can help exclude obvious causes, this is not always a straightforward process. Determining the etiology of hemolytic anemia can be particularly challenging in cancer patients given the confounding effects of chronic disease, disease progression, comorbid conditions, prior and concurrent interventions, and other concomitant events such as infections. Incorporating probability scales into clinical practice may help clinicians to assess the likelihood of DIIHA in patients treated with ICIs. One such scale is the Naranjo Adverse Drug Reaction (ADR) probability scale, a semi-quantitative tool that estimates the likelihood that a drug caused an adverse clinical event based on several indicators, including a reasonable temporal sequence after drug administration, recognized pattern of response to the suspected drug, improvement on drug withdrawal, reappearance of adverse event upon re-exposure, exclusion of alternative causes, placebo response, drug levels in body fluids or tissues, dose-response relationship, previous patient experience with the drug, and confirmation by objective evidence (32). Four case-reports were identified that used the Naranjo Adverse Drug Reaction (ADR) probability scale to assess the causality between ICI therapy and IHA, with reported scores ranging from as low as 4, indicating ‘possible’ causality, to as high as 7, indicating ‘probable’ causality (33–36).

In most cases, however, a final diagnosis often requires extensive ancillary laboratory tests. Although laboratory tests are useful for excluding other underlying conditions, there are a large number of possible tests, which may be time-consuming and expensive to perform, and the results can often be inconclusive (37). The main consideration when choosing which laboratory tests to perform should be whether the test results are expected to alter patient care decisions and guide treatment.

For instance, distinguishing between warm and cold AIHA is important because corticosteroids generally have poor efficacy in cold cases, and first-line rituximab therapy can be considered instead. In contrast, rituximab therapy is recommended after corticosteroid failure in cases of wAIHA. In line with this, response to second-line rituximab was reported in three cases of cold-type ICI-associated IHA resistant to first-line corticosteroid therapy, including one case in a patient with a history of MZL (38–40). Response to first-line rituximab (used alone, concomitantly, or sequentially with corticosteroids) was reported in three cases of cold-type ICI-associated IHA, with one case in a patient whose bone marrow evaluation was interpreted as a low-grade MZL (41–43). Nevertheless, first-line corticosteroid therapy has also been reported to be successful in cases of cold agglutinins caused by ICI therapy, with one case in a patient with bone marrow hypocellularity, histiocytic hyperplasia, and hemophagocytic macrophage infiltration (36, 44, 45). Given the general efficacy of rituximab and the toxicity associated with long-term glucocorticoid therapy, it is important to determine whether rituximab should be administered earlier in the treatment of secondary, cold-type DIIHA to minimize exposure to glucocorticoids.

Similarly, once AIHA is diagnosed, investigating for an underlying associated condition is critical because the treatment approach may differ. For instance, in patients with secondary AIHA, improvement is often achieved by treating the associated condition according to best clinical practice guidelines (22, 46). In contrast, if the associated condition appears inactive or does not meet treatment criteria, management can proceed along similar lines to that of primary AIHA and involves therapy directed at controlling autoimmunity (22, 47). However, this treatment approach is not always successful, as in the case reported by Yun et al. of a patient with a 5-year history of indolent CLL, who did not meet the criteria for treatment and developed DAT-negative AIHA after starting pembrolizumab therapy for urothelial carcinoma (48). In this case, prednisone tapering resulted in AIHA relapse and subsequent rituximab therapy caused severe infusion-related reactions. Despite no evidence suggesting bone marrow failure, active or progressive disease, targeted treatment for CLL with ibrutinib was ultimately effective in treating AIHA in this patient.

Consistent with previous recommendations for the management of irAEs (5, 29, 30, 49), this review found that the management of ICI-associated IHA involved the suspension of ICI therapy in 87 cases (94.6%), first-line corticosteroid therapy with tapering in 90 cases (97.8%), and transfusion support in 58 cases (63%). Only 9 of 23 (39.1%) patients who were rechallenged with an ICI after a temporary hold, went on to develop an irAE. This included 7 patients with ICI-associated IHA who relapsed. In contrast, irAEs and IHA relapse seemed to occur more frequently in patients in whom ICI therapy was not withheld. These results are consistent with the work of Hwang et al. (2022) who suggested that ICI rechallenge with close monitoring can be considered in patients with resolved hemolysis, especially when alternative treatment options are limited and a substantial clinical benefit from ICI therapy had been obtained (50).

Adding another layer of complexity, ICIs modulate critical pathways of immune regulation and can cause DIIHA or trigger the development or exacerbation of conditions that manifest clinically as AIHA, including myeloid neoplasms and autoimmune conditions, such as type 1 diabetes mellitus, Sjoügren’s syndrome, rheumatoid arthritis, and myasthenia gravis (3, 49, 51–53). Ghosn et al. described the case of a patient with melanoma without evidence of pre-existing autoimmunity who developed hemolytic anemia and neuro-Sjoügren’s syndrome while on pembrolizumab therapy (54). The origin of hemolytic anemia in this case was thought to be secondary to Sjoügren’s syndrome; however, the authors acknowledged that a direct attribution to pembrolizumab could not be excluded. Similarly, Nakatsuru et al. described the case of a patient with lung adenocarcinoma treated with a combination of atezolizumab, carboplatin, pemetrexed, and bevacizumab who developed AIHA and myelodysplastic syndrome (MDS) after three cycles of maintenance therapy with atezolizumab and bevacizumab, which progressed to acute myeloid leukemia (AML) 20 months after treatment initiation (53). These cases highlight the difficulty in establishing diagnostic and therapeutic algorithms for patients with cancer treated with ICIs. An integrated clinical and laboratory approach to diagnosis led by a multidisciplinary team of physicians may be more likely to integrate all confounding factors and make optimal individualized management decisions.

Having discussed the clinicopathological characteristics and features of ICI-associated IHA, the following section addresses the topic of potential risk factors for the development of ICI-associated IHA. Except for a past medical history (PMHx) of autoimmune diseases, there is currently a paucity of data regarding factors that can be used to predict which patients will develop irAEs (55). Little is known about variables that could be used to identify individuals at risk of developing DIIHA while on ICI therapy. This stands in contrast with the multiple risk factors that have been identified for the development of AIHA outside of the context of ICI therapy, which include: 1) hematolymphoid malignancies such as chronic lymphocytic leukemia (CLL), Hodgkin lymphoma (HL), non-Hodgkin lymphoma (NHL), and Waldenström macroglobulinemia; 2) autoimmune diseases such as systemic lupus erythematosus (SLE), rheumatoid arthritis (RA), Sjoügren syndrome, scleroderma, colitis, and primary biliary cirrhosis; 3) immunodeficiencies; 4) viral infections (Epstein-Barr virus [EBV], cytomegalovirus [CMV], hepatitis B and C, HIV, herpes simplex virus 1 and 2 [HSV1/2], varicella zoster virus [VZV], and more recently SARS-CoV-2) and bacterial infections (*Mycoplasma pneumoniae*, *Mycobacterium tuberculosis*); 5) allogenic transplantation (blood products, hematopoietic stem cells, and solid organs) and; 6) medications (46, 56–61).

Reports of ICI-associated IHA providing a detailed health history of patients could help uncover factors that contribute to the risk of developing this complication. Nevertheless, the usefulness of case reports and case series relies on the disclosure and completeness of patient-level information (62). As expected, detailed patient-level information is often missing in case series and population-based studies of hem-irAEs. A notable exception was the study by Leaf et al., who reported the clinical characteristics, laboratory features, response to treatment, recurrence rates, list of medications, and PMHx of 14 patients with ICI-associated IHA (25).

Notably, however, the health history of patients is also frequently missing or incomplete in case reports of ICI-associated IHA. Most identified cases typically reported demographic information (age and sex), history of present illness (HPI), physical examination (PE), and laboratory data related to ICI-associated IHA. In contrast, the PMHx, which includes all childhood and adult illnesses (e.g., allergies, asthma, infections, autoimmune disorders) and interventions (e.g., immunizations, surgeries, blood transfusions, medications), was often restricted to the primary tumor type, disease stage, and previous lines of systemic therapy.

Very few studies provided information on social determinants of health (SHx), such as personal habits (e.g., tobacco and alcohol use) or exposures (e.g., pesticides, insect bites, etc.), which may contribute to the risk of IHA development. Surprisingly, nine articles involving 12 patients did not report any information other than the HPI. Similarly, only two studies reported a family history (FHx) of blood and autoimmune disorders (34, 63).

Of the very few cases of ICI-associated IHA documenting the medication history of patients, the majority reported the concomitant use of medications that have been causally associated with the development of DIIHA. However, in none of these cases were these medications deemed to be the cause of DIHA. Only two studies conducted serological investigations to determine the causal drugs of DIIHA (64, 65). This issue is growing in importance given the increasing number of indications for ICIs in combination with cytotoxic chemotherapy, targeted agents, and other immunotherapies. Chambers et al. reported the absence of drug-dependent antibodies in blood and drug samples of atezolizumab and naproxen (a drug known to cause DIIHA via drug-dependent antibodies), excluding naproxen as the causative agent, while providing evidence that DIIHA secondary to atezolizumab occurs through a drug-independent mechanism (64). In contrast, Zhao et al. reported a case of a patient treated with nivolumab in combination with XELOX Cetuximab, in which the presence of oxaliplatin antibodies was the cause of DIIHA (65).

The high overall proportion of missing data in ICI-associated IHA reports is likely due to the omission of information that is considered irrelevant. However, deciding in advance which elements of the health history of patients are relevant and worth reporting implies that the variables associated with the risk, severity, and outcomes of patients with ICI-associated IHA are already known. Although the incompleteness of data in case reports can also be attributed to space constraints in publications, the influence of cognitive biases that strengthen the causality between ICI therapy and DIIHA cannot be discarded.

Despite the high overall proportion of missing data, this review identified several cases of ICI-associated IHA in patients with comorbidities known to be associated with AIHA, including 16 cases in patients treated for solid tumors with a PMHx of hematolymphoid tumors. This represents 17.4% of all cases, 28.6% of cases documenting the presence or absence of comorbidities, and 34.8% of all comorbid conditions. This finding suggests that patients with a medical history of hematolymphoid malignancies may be at an increased risk of developing ICI-associated IHA and may benefit from closer surveillance. AIHA is a common complication among patients with lymphoproliferative disorders. However, it has rarely been reported as a paraneoplastic event in patients with specific primary solid tumors, such as thymomas and ovarian and prostate cancers (37). Thus, the high proportion of ICI-associated IHA in patients with primary solid tumors (80 of 84 [95.2%]) other than thymomas, ovarian, or prostate cancers further strengthens the causative involvement of ICIs in the development of DIIHA.

Another potential risk factor for the development of ICI-associated IHA might be the presence of anemia prior to ICI initiation. Ten cases of ICI-associated IHA were identified in patients with pre-existing anemia (35, 45, 66), AIHA (40, 44, 67, 68), or a history of RBC alloantibodies, autoantibodies, and cold agglutinins (15, 63, 69). In this regard, the case described by Ogawa et al. of a patient with advanced NSCLC who presented with AIHA after one cycle of pembrolizumab therapy stands out because the autoimmune status of the patient was investigated before and after treatment, with positive DAT and IAT results at both time points suggesting the exacerbation of pre-existing AIHA (67).

Similarly, Jain et al. described a case of hemolytic anemia secondary to pembrolizumab in a NSCLC patient who, prior to ICI initiation, had severe anemia, a positive DAT (C3+, IgG–), and a cold agglutinin titer of 64, but no markers of hemolysis (44). However, DAT and cold agglutinin tests were not performed after ICI treatment to corroborate the exacerbation of CAS. This index case prompted the authors to conduct a retrospective study to investigate the association between the presence of autoantibodies (determined by a positive DAT) or alloantibodies prior to ICI treatment and the development of irAEs. It was found that, in contrast to a positive DAT prior to ICI treatment, the presence of alloantibodies prior to ICI was significantly associated with the development of non-hem-irAEs (44). These findings must be interpreted with caution given the small number of patients tested for autoantibodies or alloantibodies at baseline. As stated by the authors, these results may also reflect the selection of a population with a worse overall health status, given that RBC antibody testing is more likely to be performed in patients requiring blood transfusion.

The formation of autoantibodies against RBC antigens has been reported to occur as a complication of blood transfusions, usually in association with alloimmunization, suggesting that immune responses against foreign antigens can expand to self-antigens via several mechanisms including molecular mimicry, immune complex formation, and molecular adjuvancy (70). Thus, exposure to foreign RBC antigens and sensitization to other self-antigens may underlie the increased risk of non-hematological irAEs in the patient population described by Jain et al. In line with this, four cases of ICI-associated IHA in patients with a history of blood transfusions were identified (15, 34, 43, 69), including the case report by Kong et al. (2009), who described one of the earliest cases of nivolumab-associated IHA in an 85-year-old male patient with metastatic melanoma and a history of RBC alloantibodies and autoantibodies, which had been detected 3 years before any treatment for metastatic disease prior to the transfusion of 7 units of packed RBCs (15).

The potential of ICIs to exacerbate or unmask AIHA, as well as other autoimmune conditions that manifest clinically as AIHA (55, 71), raises the question of the utility of screening asymptomatic patients for autoimmunity before ICI therapy initiation. From a research standpoint, baseline screening might help clarify several aspects about the nature of ICI-associated IHA and its relationship with other autoimmune conditions, including whether in patients treated with ICIs, IHA arises *de novo* or from pre-existing AIHA, and thus, to better estimate the risk of ICI-associated IHA. Clinically, however, the magnitude of the benefits, harms, and burdens stemming from baseline screening should be considered. Baseline screening would be justified if screening-informed treatment resulted in a favorable benefit-to-harm balance compared with treatment in the absence of screening results.

There are several arguments against recommending universal baseline screening for autoantibodies. First, although autoimmune markers (e.g., anti-nuclear antibodies [ANA], rheumatoid factor [RF], IgG, IgM, anti-thyroid peroxidase antibodies (anti-ThyPeroxAb), anticardiolipin antibodies [ACL], and lupus anticoagulant [LAC]) are usually present in patients with autoimmune disorders (e.g., scleroderma, Sjögren’s syndrome, myositis, SLE, RA, myasthenia gravis, and ITP), they may also be detected in patients with non-autoimmune diseases and in healthy individuals who may never develop an autoimmune disease (72, 73). Consequently, test results alone are neither diagnostic nor predictive of autoimmune disease. Second, despite the exclusion of patients with autoimmune diseases from clinical trials involving ICIs, data from several retrospective studies suggest that ICIs are safe for patients with cancer and autoimmune diseases (55, 74–79). Together, these studies show that only a minority of cancer patients have an exacerbation of their autoimmune disease, with an increased risk of developing *de novo* irAEs, which are generally manageable and rarely require permanent ICI discontinuation.

Thus, the available evidence does not support a contraindication for ICI therapy in individuals with positive test results or formally diagnosed with an autoimmune disease during screening. Instead, implementation of universal screening in a population at average risk for autoimmunity may only cause management dilemmas for physicians and undue confusion, anxiety, and inconvenience for patients with cancer, who already undergo a multitude of diagnostic and therapeutic interventions. However, a careful health history assessment prior to treatment, with particular attention paid to the PMHx and FHx of autoimmune diseases and their most frequently associated signs and symptoms, may help select patients who are more likely to benefit from advanced baseline testing. In line with this, Martins et al. recently proposed a personalized risk-based surveillance strategy that combines mandatory pre-treatment clinical and biological assessments for patients with autoimmune diseases (3).

The findings of this review must be interpreted with caution, given the inherent limitations of retrospective studies, case reports, and case series on which this review was based. As reports of rare conditions, including ICI-associated IHA, are subject to reporting and publication bias (i.e., unusual features are more likely to be reported and published), they may not be representative of the wider population and cannot be used to infer the true frequency of co-occurring conditions or their contribution to the risk of ICI-associated IHA.

Another arguable weakness is that the inclusion of studies in this review was not based on reporting quality or completeness. Rather than excluding publications, we decided to identify important information that is frequently omitted or inadequately reported because, until more robust studies are conducted and higher-level evidence becomes available, retrospective studies, case reports, and series will remain the primary sources of information for rare irAEs, such as ICI-associated IHA.

Most case reports focus on positive findings, while a limited number document the absence of signs, symptoms, or specific elements of the adult health history known to be associated with the risk of AIHA and DIIHA. The high proportion of missing data is a major source of information bias, which can inflate the relevance of certain health history elements or minimize the contribution of other factors that could be associated with the risk, severity, and outcomes of patients with ICI-associated IHA. For example, cases of patients with a positive history of smoking or previous transfusions may have been more likely to report this information. Thus, it is uncertain whether the high proportion of ICI-associated IHA cases in patients who smoked or had previous transfusions is clinically relevant, or merely a reflection of the limited number of reports documenting this information.

An important final consideration is that our criteria for defining ICI-associated IHA did not require ICI therapy to be considered the most likely etiology of IHA. Our definition was broadened to include cases that illustrate the multifactorial etiology and etiologic heterogeneity of ICI-associated IHA and the difficulty in establishing causality (42, 53, 54). As previously mentioned, immunomodulatory agents, such as ICIs, have uncovered new settings of immune dysregulation that may cause the development, reactivation, or exacerbation of AIHA, as well as other conditions, including autoimmune conditions that manifest clinically as AIHA, making it challenging to differentiate between primary AIHA, secondary AIHA, and DIIHA.

## 5 Conclusions

To the best of our knowledge, this is the largest systematic review of population-based studies, case-reports and case-series of IHA associated with anti-CTLA-4 and anti-PD-(L)1 therapy. ICI-associated IHA has a complex multifactorial etiology that includes intrinsic and extrinsic factors that contribute to the breakdown of immune tolerance and determine whether an individual will develop this complication. This review identified a high proportion of cases of ICI-associated IHA in patients with a PMHx of hematolymphoid tumors, hypertension, pre-existing anemia/AIHA, diabetes, multiple primary solid tumors, and a history of previous transfusions, suggesting that these conditions might increase the risk of ICI-associated IHA. In the absence of a predictive tool to individually estimate the risk of ICI-associated IHA, a comprehensive list of frequently reported conditions in cases of ICI-associated IHA may help to select patients who could benefit from closer monitoring. Despite the recognition that virtually all diseases arise from the interaction of multiple causes, most studies on AIHA, DIIHA, and ICI-associated IHA have focused on identifying single causes and risk factors. Thus, the next step is to move towards a multifactorial approach to the study of ICI-associated IHA. Although the rarity of ICI-associated IHA may hinder the development of large-scale prospective studies, future research should aim to identify high-risk individuals and provide evidence-based recommendations for surveillance, diagnosis, and management.

## Data Availability

The original contributions presented in the study are included in the article/**Supplementary Material**. Further inquiries can be directed to the corresponding author.
