# Supplementary material for: Immune hemolytic anemia associated with the use of immune checkpoint inhibitors: a scoping review
Source: Front Immunol. 2025 Jul 3;16:1586426. doi: 10.3389/fimmu.2025.1586426 (PMC12268707; doi:10.3389/fimmu.2025.1586426)
Supplement: Supplementary file 1 [file DataSheet1.pdf]

Supplementary Table 1. Summary of information extracted from reports of ICI-associated IHA.

Reference	Age/ Sex	Tumor type	Triggering ICI	Onset (cycles)	Comorbidities/ Medical history	Diagnosis & workup	IHA treatment	Outcome	Rechallenge	Recurrence after rechallenge	Fatal
Simeone *et al.* 2014	68 y/o Female	Melanoma	2nd: Ipi 3 mg/kg<br>1st: Temozolomide	3	- NR	AIHA<br>↓Hb<br>↑LDH<br>↑BILI<br>↑Retic count<br>- Iron<br>↑Ferritin<br>- DAT: (+)<br>- Blood smear: Spherocytes<br>- WBCs/PLTs: Normal<br>ANAs: 1:2560 (speckled)	- ICI discontinued: Yes<br>- MPL 125 mg BID ×1 week<br>- Washed RBCs	CR	No	N/A	No
Lott *et al.* 2015	67 y/o Male	NSCLC	3rd: Pembro 10 mg/kg Q2W<br>2nd: Peme<br>1st: GEM/Carbo	18	- 70 pack-years of smoking<br>- Left lower extremity DVT<br>- Drugs: enoxaparin	Evans syndrome<br>↓Hb<br>↓Hematocrit<br>↑Total BILI<br>↑LDH<br>↓Haptoglobin<br>↑Retic count: 239<br>- Blood smear: Microspherocytes & thrombocytopenia<br>- DAT: IgG+<br>- IAT: IgG+<br>↓PLTs<br>- Liver enzymes & creatinine: Normal<br>- Serologic tests: negative for infectious & rheumatologic diseases	- ICI discontinued: Yes<br>- Pred, azathioprine, CTX, IVIG, erythropoietin IV, transfusion support, then Pred 105 mg PO & CTX 100 mg, Pred tapering<br><br>Salvage:<br><br>- Rituximab Q1W ×4, high-dose Pred with 24-week taper	CR	No	N/A	No
Kong *et al.* 2016	85 y/o Male	Melanoma	3rd: Nivo 3 mg/kg Q2W<br>2nd: Ipi 3 mg/kg Q3W, 4 cycles<br>1st: Dacarbazine, 6 cycles	5	- Bioprosthetic mitral valve replacement<br>- h/o Blood transfusion<br>- Allo-Abs<br>- Prior DAT+	AIHA<br>↓Hb<br>↑LDH<br>↑BILI<br>↑Retic count<br>↓Haptoglobin<br>- DAT: IgG+<br>- Blood smear: polychromasia, agglutination, spherocytes<br>- WBCs/PLTs: Normal	- ICI discontinued: Yes<br>- High-dose Pred<br>- 2 units RBCs	Response	No	N/A	No
Nair *et al.* 2016	52 y/o Female	Melanoma	3rd: Pembro<br>2nd: Ipi<br>1st: Adjuvant RT & high-dose IV IFN	3	- Prior treatment with IFN ×1 month<br>- Autoimmune hepatitis on Ipi therapy	wAIHA & PRCA<br>↓Hb<br>↑LDH<br>↑BILI<br>↓Retic count	- ICI discontinued: Yes<br>- Glucocorticoids<br>- IVIG	Response	No	N/A	No

						↓Haptoglobin<br>- DAT: IgG+, C3b–<br>- Blood smear: Anisocytosis<br>- Bone marrow<br>- WBCs/PLTs: Normal					
Palla *et al.* 2016	70 y/o Male	LUAD	2nd: Nivo 3 mg/kg Q2W<br>1st: Carbo/Peme, 3 cycles	2	- NR<br>- Not on any other AIHA- associated drugs	AIHA<br>↓Hb<br>↑LDH<br>↑Retic count<br>- DAT: IgG-, C3+<br>- Blood smear: Spherocytes, polychromasia, nucleated RBC<br>- Warm agglutination: (+)<br>- Cold agglutination: (–)	- ICI discontinued: Yes<br>- Pred 1 mg/kg PO QD<br>- Pred 1.5 mg/kg QD	No Response	No	N/A	Yes
Schwab *et al.* 2016	82 y/o Male	Cutaneous SCC	3rd: Nivo 3 mg/kg Q2W<br>1st Cis+RT<br>2nd: Cetuximab + Dtx<br>- For CLL<br>2nd: Ibrutinib<br>1st: Rituximab, fludarabine & CTX	8	- CLL<br>- HTN<br>- Prostate cancer (radical prostatectomy)<br>- Prior Hb & PLT count decrease	AIHA<br>↓Hb<br>- Irregular Abs: (+)<br>- Warm Abs: (+)<br>- DAT: IgG+, C3+<br>- LDH: NR<br>- BILI: NR<br>- Retic count: NR<br>- Haptoglobin: NR<br>- Blood smear: NR	- ICI discontinued: Yes<br>- PSL 80 mg PO QD	CR	No	N/A	No
Khan *et al.* 2017	43 y/o Female	Melanoma	1st: Ipi + Nivo<br>WBRT	2	- Regular use of NSAIDs<br>- HT<br>- Obesity	AIHA<br>↓Hb<br>↑LDH<br>↑BILI<br>↑Retic count<br>↓Haptoglobin<br>- DAT: IgG+, C3d+<br>- WBCs<br>- PLTs<br>- Blood smear: spherocytes, polychromasia	- ICI discontinued: Yes<br>- MPL 1 g IV QD ×3, then Pred 1 mg/kg PO QD<br>- Multiple blood transfusions<br><br>After rechallenge & recurrence<br><br>- Rituximab 375 mg/m^2 IV ×4	Response	Yes	Yes	No
Ramos *et al.* 2017	71 y/o Male	Melanoma	2nd: Ipi 3 mg/kg Q3W, adjuvant RT to the groin & pelvic region<br>1st: Dacarbazine 250 mg/m^2 ×5 days every 28 days	4	- No medications, respiratory, urinary, or gastrointestinal infections in the preceding 4 months	AIHA<br>↑LDH<br>↑BILI<br>↑Retic count<br>↓Haptoglobin<br>- DAT: IgG+, C3d+<br>- WBCs<br>- PLTs<br>- LFT<br>- Anti-DNA: (–)<br>- ANAs: (–)	- ICI discontinued: Yes<br>- PSL 1 mg/kg PO QD ×8 weeks	CR	No	N/A	No

						- UPEP & SPEP: Normal<br>- Bone marrow: erythroid hyperreactive marrow					
Tardy *et al.* 2017	75 y/o Female	Hodgkin lymphoma	3rd: Nivo 3 mg/kg Q2W<br>2nd: Bendamustine & brentuximab vedotin<br>3D-CRT to the mediastinum<br>1st: ABVD	2	- "Without any significant medical history or comorbidities"	AIHA<br>↓Hb<br>↑LDH<br>↑BILI<br>↑Retic count<br>- DAT: IgG+<br>- IAT: (+)<br>- WBCs<br>- PLTs<br>- LFT	- ICI discontinued: Yes<br>- Pred 2 mg/kg PO QD then 1 mg/kg/day ×3 months<br>- 2 units RBCs	CR	Yes	No	No
Le Burel *et al.* 2017	72 y/o Female	Melanoma	Anti-PD-1 (not specified) previously treated with Ipi	5	- h/o cold agglutinins	AIHA<br>↓Hb<br>- Warm agglutinins<br>- Normal blood marrow agglutinins aspirate smears	- ICI discontinued: Yes<br>- Corticosteroids 2 mg/kg QD	Response	No	N/A	No
Deltombe *et al.* 2017	73 y/o Male	Melanoma	1st: Nivo 3 mg/kg Q4W	2	- Kidney transplant 15 months prior to cancer diagnosis<br>- Upon cancer diagnosis immunosuppression was reduced, & tacrolimus was switched to low- dose everolimus<br>- Allograft rejection	AIHA & severe thrombocytopenia<br>- DAT: C+<br>↓PLTs<br>- Donor-specific Abs: (–)	- ICI discontinued: Yes<br>- High-dose steroids	Response	No	N/A	No
Algaze *et al.* 2018	60 y/o Male	LUAD	For LUAD:<br>3rd: Nivo<br>2nd: Carbo/Peme ×4 cycles then maintenance with Peme<br>1st: Cis/Dtx + RT then Dtx<br>- Palliative radiation<br><br>For CLL:<br>3rd: Bendamustine<br>2nd: Ibrutinib then ofatumumab<br>1st: Fludarabine, mitoxantrone, dexamethasone & rituximab	21	- CLL<br>- IVIG for hypogamma globulinemia<br>- DMT2<br>- DAT+ for warm IgG auto-Abs prior to treatment<br>- Blood transfusion	AIHA<br>↓Hb<br>↑LDH<br>↑BILI<br>↑Retic count<br>↓Haptoglobin<br>- DAT: IgG+, C–, anti-Jk^a+<br>- Fibrinogen: 404<br>- WBCs<br>- PLTs<br>- Blood smear: spherocytes, reticulocytosis	- ICI discontinued: Yes<br>- MPL 80 mg IV QD<br>- 7 units RBCs<br>- B_9 supplementation	Response	Yes	No	No
Ogawa *et al.* 2018	82 y/o Male	LUAD	2nd: Pembro 200 mg Q3W<br>1st: Chemoradiation	1	- HT & chronic anemia<br>- DAT+ & IAT+ prior to Pembro therapy	AIHA (exacerbation)<br>↓Hb<br>↑LDH<br>↑BILI<br>↑Retic count<br>↓Haptoglobin<br>- DAT: (+)<br>- IAT: (+)<br>- LFT: Normal<br>↑WBCs<br>↑PLTs	- ICI discontinued: Yes<br>- Pred 60 mg PO QD<br>- Blood transfusions	Response	No	N/A	No

						- Blood smear: spherocytes<br>- Bone marrow: hyperplasia & absence of tumor.					
Shaikh *et al.* 2018	78 y/o Male	LUAD	3rd: Nivo<br>Multiple subsequent lines including single- agent Dtx, GEM & vinorelbine<br>2nd: Carbo/Pacli/Beva<br>1st: Peme/Carbo	39	- Prostate adenocarcinoma (brachytherapy)	wAIHA<br>↓Hb<br>↑LDH<br>↑BILI<br>↑Retic count<br>↓Haptoglobin<br>- DAT: IgG+, C3–<br>- IAT: (+)<br>- WBCs/PLTs: Normal<br>- Flow cytometry<br>- Immunofixation, electrophoresis, SPEP & cold agglutinin: Normal<br>- Bone marrow: inconclusive for MDS	- ICI discontinued: Yes<br>- Low- dose steroid<br>- Pred 1 mg/kg PO QD, rituximab ×4<br>- Periodic blood transfusions<br>- Erythropoietin injections 40,000 units Q1W	Response	No	N/A	No
Sun *et al.* 2018	58 y/o Female	Melanoma	2nd: Pembro & IL-10 after disease progression	1	- NR	AIHA (DAT–)<br>↓Hb<br>↑LDH<br>↑BILI<br>↓Retic count<br>↓Haptoglobin<br>RBC allo-Abs (–)<br>DAT: (–)<br>- Blood smear: anisocytosis, spherocytes<br>- Normal B_{12} & B_9<br>- Parvovirus B19 (–)<br>- EBV: (–)<br>- Bone marrow: benign changes	- ICI discontinued: Yes<br>- 5 units RBCs<br>- MPL 1 mg/kg IV QD ×3<br>, then Pred 1 mg/kg PO QD ×2 weeks	Response	Yes	No	No
Sun *et al.* 2018	62 y/o Male	SCLC	2nd: Pembro & RT<br>1st: Carbo/etoposide, pro-phylactic cranial irradiation (5 cycles)	1	- NR	AIHA (DAT–)<br>↓Hb<br>↑LDH<br>↑BILI<br>↑Reticulocytes<br>↓Haptoglobin<br>- DAT: (–)	- ICI discontinued: Yes<br>- Pred 1 mg/kg PO QD	Response	No	N/A	No
Sun *et al.* 2018	64 y/o Male	Melanoma	1st: Ipi & Nivo	2	- Other irAEs<br>ITP<br><br>- HTN<br>- Diverticulosis<br>- Colonic polyps<br>- HCL<br>- COPD	AIHA (DAT–)<br>↓Hb<br>↑LDH<br>↑Ferritin<br>Normal B_{12} & B_9<br>↓Reticulocytes<br>↓Haptoglobin<br>- DAT: (–)<br>- RBC allo-Abs (–)	- ICI discontinued: Yes<br>- 2 units RBCs (×2)<br>- Pred 1 mg/kg PO QD ×38<br>- Pred 2 mg/kg PO QD or MPL IV<br>- IVIG 1 g/kg/day ×2	Response	Yes	N/A	No

						↓PTLs<br>- WBCs/PLTs: Normal<br>- Blood smear: anisopoikilocytosis, microspherocytes, few bands, rare metamyelocytes					
Robilliard *et al.* 2018	79 y/o Female	Melanoma (Choroidal)	4th: Pembro Q3W ×4, then sorafenib<br>3rd: Chemoembolization ×4<br>2nd: Complete removal of the eye<br>1st: Proton therapy	4	- NR	AIHA & polymyalgia rheumatica<br>↓Hb<br>- Total BILI: 7 µmol/L<br>- LDH: 235 UI/L<br>- Haptoglobin: 0.66 g/L<br>- Reticulocytes: 195 × 10^9/L<br>- DAT: IgG+, C3+<br>- MCV: 106 fL<br>- CRP: 68 mg/L	- ICI discontinued: Yes<br>- Corticosteroids 1 mg/kg	CR	No	N/A	No
Ghosn *et al.* 2018	69 y/o Female	Melanoma	- Pembro + T-VEC	11	- No known h/o autoimmune disorders or neurologic manifestations prior to immunotherapy	Sjören syndrome & hemolytic anemia after relapse<br>- Paresthesia<br>- Absence of sensory nerve action potentials<br>- MRI: Enhancement of the right trigeminal Gasser's ganglia & its mandibular branch<br>- CSF analysis: ↑ Protein, pleocytosis, cultures (–)<br>- CSF PCR: viral (–)<br>- Infectious panel: (–)<br><br>After relapse<br>↓Hb<br>↓ Haptoglobin<br>↑ LDH<br>↑ Total BILI<br>↓ Lymphocytes (0.8 G/L)<br>↑CRP<br>↑ESR<br>↑ Total IgG<br>- ANAs: (+)<br>- ANCAs: (–)<br>- Paraneoplastic neurologic syndrome Abs: (–)<br>- Accessory salivary gland biopsy: abnormal interstitial sclerosis with a focus of >50 lymphocytes/4 mm^2	- ICI discontinued: Yes<br>- MPL 1 g QD (× 5)<br><br>After relapse<br>1st: MPL 1 g QD, IVIG 0.4 g/kg QD (×5), acyclovir<br>2nd: CTX 15 mg/kg, Pred 60 mg PO QD (×1)<br>- Rituximab 375 mg/m^2 Q1W ×6 months	CR	No	N/A	No
Hasanov *et al.* 2018	89 y/o Female	Urothelial carcinoma	2nd: Nivo 6 mg/kg Q4W<br>1st: GEM & Carbo 2 cycles	7	- h/o breast cancer<br>- h/o MZL<br>- HT	Pancytopenia → CAS<br>- Cold agglutinin (+, >64)<br>↓Hb	- ICI discontinued: Yes<br>- 2 units RBCs<br>- Pred 1 mg/kg PO QD	No Response	No	N/A	No

						↑LDH<br>↑BILI<br>- Haptoglobin: Normal<br>- Blood smear: Anisocytosis, few hypochromic RBCs, abundant microspherocytes, roleaux, agglutination<br>- DAT: IgG–, C3d+<br>- WBCs<br>- PLTs<br>- TFTs: normal<br>↑B_9<br>- B_{12}: Normal<br>- Copper: Normal<br>- Bone marrow: No evidence of malignancy	- 5 units RBCs<br><br>- Rituximab 1g Q2W IV (×4)	CR			
Johnstone *et al.* 2019	73 y/o Male	LUAD	1st: Pembro	13	- COPD<br>- HTN<br>- Atrial fibrillation<br>- Previous bilateral phacoemulsification.<br>- Drugs associated with AHIA	AIHA<br>↓Hb<br>↑LDH<br>↑BILI<br>↑Retic count<br>↓Haptoglobin<br>- DAT: (+)<br>↑WBCs/PLTs	- ICI discontinued: Yes<br>- PSL 1 mg/kg + omeprazole & B_9 supplementation<br>- 2 units RBCs	CR	No	N/A	No
Delanoy *et al.* 2019	58 y/o Female	Melanoma	7th: Pembro<br>- Previous treatment: NR	2	- NR	AIHA<br>- DAT: IgG+<br>- Bone marrow: Aspiration was near normal	- ICI discontinued: Yes<br>- Pred 80 mg/kg PO QD, 4 units RBCs, EPO<br>- Resolved	CR	No	N/A	No
Delanoy *et al.* 2019	72 y/o Male	NSCLC	- Nivo<br>- Previous treatment: NR	NR	- NR	AIHA<br>- DAT: C3d+<br>- Cold Ab: (+)<br>- Cold agglutinin: (–)<br>- Bone marrow: Not done	- ICI discontinued: Yes<br>- Pred 1 mg/kg PO QD, rituximab, 4 units RBCs<br>- Not resolved	Not Resolved	No	N/A	No
Delanoy *et al.* 2019	56 y/o Male	Melanoma	3rd: Pembro<br>- Previous treatment: NR	21	- NR	AIHA<br>- DAT: C3d+<br>- Bone marrow aspiration: Erythroblasts 20%, granular cells 49%, lymphocytes 30%<br>- Bone marrow biopsy: Normal cellularity, no malignant infiltration	- ICI discontinued: Yes<br>- Pred 2 mg/kg PO QD, 2 units RBCs<br>- Resolved	CR	No	N/A	No
Delanoy *et al.* 2019	69 y/o Male	Melanoma	2nd: Nivo<br>- Previous treatment: NR	2	- NR	AIHA<br>- DAT: C3d+<br>- Cold agglutinin: (–)<br>- PNH: (–)<br>- Bone marrow aspiration: normal cellularity, no dysplasia, no malignant infiltration	- ICI discontinued: Yes<br>- Pred 2 mg/kg PO QD, 3 units RBCs<br>- Resolved	CR	No	N/A	No

Delanoy *et al.* 2019	78 y/o Male	Melanoma	1st: Pembro	6	- B-cell CLL	AIHA<br>- DAT: C3d+<br>- Bone marrow aspiration: B-cell malignant reliable with underlying B-cell CLL	- ICI discontinued: Yes<br>- Pred 2 mg/kg PO QD, IgIV, rituximab, 2 units RBCs<br>- Not resolved	Not Resolved	No	N/A	No
Delanoy *et al.* 2019	29 y/o Female	RCC (papillary)	3rd: Atezo<br>- Previous treatment: NR	1	- NR	AIHA<br>- DAT: C3d+<br>- Cold Ab: (+)<br>- Cold agglutinin: 1/256<br>- Bone marrow: Not done	- ICI discontinued: Yes<br>- Pred 2 mg/kg PO QD, 2 units RBCs, rituximab<br>- Not resolved	Not Resolved	No	N/A	No
Delanoy *et al.* 2019	77 y/o Male	RCC (clear cell)	2nd: Nivo<br>Previous treatment: NR	2	- B-cell CLL	AIHA<br>- DAT: C3d+\<br>Cold Ab: (+)	- ICI discontinued: Yes<br>- Pred 1 mg/kg PO QD, 4 units RBCs, rituximab<br>- Resolved	CR	No	N/A	No
Delanoy *et al.* 2019	60 y/o Male	NSCLC	3rd: Nivo<br>- Previous lines: NR	2	- NR	AIHA<br>- DAT: IgG+<br>- Bone marrow: Not done	- ICI discontinued: Yes<br>- MPL IV, then Pred 1 mg/kg PO QD, 8 units RBC<br>- Resolved	CR	Yes	No	No
Delanoy *et al.* 2019	89 y/o Female	NSCLC	4th: Nivo<br>- Previous lines: NR	3	- NR	AIHA<br>- DAT: IgG+<br>Anti-extractable nuclear antigen: (+), Scl70 type<br>- Bone marrow: Rich erythroblastic lineage, no signs of dyserythropoiesis or malignant cells.	- ICI discontinued: Yes<br>- Pred PO, rituximab, 1 unit RBCs<br>- Resolved	CR	No	N/A	No
Ni *et al.* 2019	67 y/o Male	Melanoma	1st: Pembro	8	- CLL	AIHA & pancytopenia<br>↓Hb<br>↑Retic count<br>↓PLTs<br>↓Neutrophils<br>↓WBCs<br>- DAT: (+)<br>- Blood smear: normocytic, no spherocytes<br>- Bone marrow: consistent with CLL, reduced erythropoiesis	- ICI discontinued: Yes<br>- MPL IV & RBCs<br>- Pred 1 mg/kg/d	Response	No	N/A	No
Tanios *et al.* 2019	52 y/o Male	LUAD	3rd: Nivo<br>2nd: Dtx/Peme<br>1st: Carbo/Pacli & RT	24	- Regular NSAIDs use<br>- Acetaminophen as needed, albuterol & montelukast	wAIHA<br>↓Hb<br>↑LDH<br>↑BILI<br>↑Retic count<br>- Haptoglobin<br>- DAT: warm IgG+, C3–<br>- Blood smear: spherocytes polychromasia<br>- Coagulation tests: Normal	- ICI discontinued: Yes<br>- MPL equivalent to 1.5mg/kg of Pred<br>- 3 units of the least incompatible blood	No Response	No	N/A	Yes

						- Urine: Amber colored with urobilinogen					
Leaf *et al.* 2019	85 y/o Male	Melanoma	1st: Pembro 2 mg/kg Q3W	2	- MZL (not treated)<br>- DMT2<br>- Drug list provided but not deemed to be the cause of AIHA	AIHA<br>↓Hb<br>↑LDH<br>↓Haptoglobin<br>- DAT: (–)	- ICI discontinued: Yes<br>- Pred 1 mg/kg PO QD<br>- Rituximab 375 mg/m^2/week IV ×4	CR	No	N/A	No
Leaf *et al.* 2019	48 y/o Male	Melanoma	1st: Ipi 3 mg/kg + Nivo 1 mg/kg both Q3W	3	- None<br>- Drug list provided but not deemed to be the cause of AIHA	AIHA<br>↓Hb<br>↑LDH<br>↓Haptoglobin<br>- DAT: IgG+, C3+	- ICI discontinued: Yes<br>- MPL 100-200 mg IV ×4<br>- Pred 60 mg PO QD	CR	No	N/A	No
Leaf *et al.* 2019	67 y/o Female	Melanoma	1st: Ipi 3 mg/kg + Nivo 1 mg/kg both Q3W	4	- None<br>- Drug list provided but not deemed to be the cause of AIHA	AIHA<br>↓Hb<br>↑LDH<br>↓Haptoglobin<br>- DAT: IgG+, C3–	- ICI discontinued: Yes<br>- Pred 60 mg PO QD	CR	Yes	No	No
Leaf *et al.* 2019*	68 y/o Male	Melanoma	1st: Pembro 2 mg/kg Q3W + indoximod	12	- Leukopenia<br>- Drug list provided but not deemed to be the cause of AIHA	AIHA<br>↓Hb<br>↑LDH<br>↓Haptoglobin<br>- DAT: IgG+, C3–	- ICI discontinued: Yes<br>- Pred 70 mg PO QD	CR	Yes	No	No
Leaf *et al.* 2019	18 y/o Male	Melanoma	1st: Ipi 3 mg/kg + Nivo 1 mg/kg both Q3W	4	- None<br>- Drug list provided but not deemed to be the cause of AIHA	AIHA<br>↓Hb<br>↑LDH<br>↓Haptoglobin<br>- DAT: (–)	- ICI discontinued: No<br>- MPL 100 mg IV ×1<br>- Pred 60 mg PO QD<br>- Pred 60 mg PO QD after pyrexia	Response	N/A	Yes	No
Leaf *et al.* 2019	47 y/o Male	Melanoma	Ipi 3 mg/kg Q3W<br>Prior: pINF, IL-2 with TILs & TBI	1	- HTN<br>- HLD<br>- Drug list provided but not deemed to be the cause of AIHA	AIHA<br>↓Hb<br>↑LDH<br>↓Haptoglobin<br>- DAT: NR	- ICI discontinued: No<br>- Dexamethasone 10 mg IV BID<br>- Dexamethasone 6 mg PO QD due to brain metastases	Response	N/A	Yes	No
Leaf *et al.* 2019	59 y/o Female	NSCLC	- Nivo 3 mg/kg Q2W<br>- priorly treated with multiple agents	12	- MZL (in remission)<br>- Drug list provided but not deemed to be the cause of AIHA	AIHA<br>↓Hb<br>↑LDH<br>↓Haptoglobin<br>- DAT: IgG+, C3+	- ICI discontinued: Yes<br>- Pred 100 mg PO QD, IVIG 0.5 g/kg QD ×2<br>After relapse<br>- Pred 60 mg PO QD, rituximab 375 mg/m^2/ week IV ×4, IVIG 1 g/kg QD ×2	Response	No	N/A	No
Leaf *et al.* 2019	63 y/o Female	NSCLC	2nd: Nivo 3 mg/kg Q2W<br>1st: Carbo/Pacli & RT)	4	- CLL (not treated)<br>- DMT2<br>- Drug list provided but not deemed to be the cause of AIHA	AIHA<br>↓Hb<br>↑LDH<br>↓Haptoglobin<br>- DAT: IgG+, C3–	- ICI discontinued: Yes<br>- MPL 60 mg IV ×1, Pred 60 mg PO QD	Response	No	N/A	No
Leaf *et al.* 2019	33 y/o Female	AML	3rd: Nivo 3 mg/kg Q2W<br>2nd: FLAG- IDA<br>1st: Chemotherapy	1	- None<br>- Drug list provided but not deemed to be the cause of AIHA	AIHA<br>↓Hb<br>↑LDH<br>↓Haptoglobin	- ICI discontinued: Yes<br>- MPL 1.25 mg/kg IV QD ×3, Pred 1.5 mg/kg PO QD	CR	No	N/A	No

						- DAT: (–)					
Leaf *et al.* 2019	85 y/o Female	Melanoma	1st: Pembro 2 mg/kg Q3W	10	- DMT2<br>- Breast cancer<br>- Drug list provided but not deemed to be the cause of AIHA	AIHA<br>↓Hb<br>↑LDH<br>↓Haptoglobin<br>- DAT: IgG+, C3–	- ICI discontinued: Yes<br>- Pred 60 mg PO QD<br>After relapse<br>- Pred 50 mg PO QD, IVIG 1 g/kg ×2, rituximab 375 mg/m² IV ×4, azathioprine 150 mg PO QD	Response	No	N/A	No
Leaf *et al.* 2019	69 y/o Female	Colorectal	- Pembro 200 mg Q3W<br>- priorly treated with multiple agents	1	- HTN<br>- Drug list provided but not deemed to be the cause of AIHA	AIHA<br>↓Hb<br>↑LDH<br>Haptoglobin: 7 mg/dL<br>- DAT: IgG+, C3+	- ICI discontinued: Yes<br>- Pred 60 mg PO QD	CR	No	N/A	No
Leaf *et al.* 2019	67 y/o Male	Melanoma	1st: Ipi 2 mg/kg + Nivo 1 mg/kg both Q3W	2	- DVT<br>- Drug list provided but not deemed to be the cause of AIHA	AIHA<br>↓Hb<br>↑LDH<br>↓Haptoglobin<br>- DAT: (–)	- ICI discontinued: Yes<br>- Dexamethasone 12 mg IV ×1, pred 1 mg/kg PO QD	CR	Yes	No	No
Leaf *et al.* 2019	55 y/o Male	Melanoma	Pembro 2 mg/kg Q3W<br>- priorly treated with multiple agents	1	- CLL<br>- ITP<br>- Drug list provided but not deemed to be the cause of AIHA	AIHA<br>↓Hb<br>↑LDH<br>- Haptoglobin: 2 mg/dL<br>- DAT: IgG+, C3+	- ICI discontinued: Yes<br>- Pred 1 mg/kg PO QD	CR	Yes	No	No
Leaf *et al.* 2019	71 y/o Female	NSCLC	1st: Pembro 200 mg Q3W	4	- None<br>- Drug list provided but not deemed to be the cause of AIHA	AIHA<br>↓Hb<br>↑LDH<br>↓Haptoglobin<br>- DAT: (–)	- ICI discontinued: No<br>- Dexamethasone 4 mg PO BID	Response	N/A	No	No
Williams *et al.* 2019	81 y/o Female	Melanoma	1st: Pembro 140 mg Q3W increased to 150 mg 21 months later	≈35 (2 yrs)	- Pseudomonas bacteremia	AIHA & cholangitis<br>↓Hb<br>↑ALP<br>↑BILI<br>↑LDH<br>↑Retic count<br>- DAT: IgG+, C3d+<br>- Blood smear: agglutination<br>- Cholangiopancreatography: acalculous cholecystitis<br>- Liver MRI: diffuse gallbladder thickening, intra- & extrahepatic biliary thickening	- ICI discontinued: Yes<br>- IV Antibiotics<br>- IV MPL × 4 days<br>- 12 units RBCs	CR	No	No	No
Okawa *et al.* 2019	78 y/o Male	LUSC	1st: Pembro 200 mg	1	- Slight macrocytic anemia<br>- Several episodes of agglutination	AIHA & HLH<br>- Cold hemagglutinin (+)<br>↓Hb<br>↑LDH<br>↑BILI	- ICI discontinued: Yes<br>- PSL 25 mg<br>- Steroid pulse therapy with antibiotics	Response	No	N/A	No

						↑Retic count<br>- DAT: (+)<br>↑Ferritin<br>↑IL-2<br>- EBV: IgG+, IgM–<br>- ANAs: (+)<br>- Bone marrow: hypocellular with histiocytic hyperplasia, hemophagocytic macrophage infiltration<br>- CT: splenomegaly					
Dutertre *et al.* 2019	26 y/o Female	RCC (papillary)	3rd: Atezo<br>2nd: Cabozantinib<br>1st: Axitinib (anti-VEGF)	1	- Severe chronic inflammatory syndrome<br>- CRP 200 mg/L prior to treatment	CAD<br>- Cold agglutinin (+, 1/256)<br>↓Hb<br>↑LDH<br>- DAT: IgG–, C3d+<br>- Blood smear: No schistocytes<br>- WBCs/PLTs: Normal<br>↑CRP	- ICI discontinued: Yes<br>- MPL 500 mg IV QD ×3 days - Pred 1.5 mg/kg PO QD<br>After relapse<br>- Rituximab 1g IV Q2W ×2	Response	No	N/A	No
Olson *et al.* 2020	29 y/o Male	Melanoma	2nd Ipi 3 mg/kg + Nivo 1 mg/kg both Q3W<br>1st Sx (excision of auricular atypical melanocytic neoplasm)	4	- 1 month prior to referral: Pruritus, ↑ALT, ↑AST, ↑ALP<br>- Levothyroxine for immune- induced HT (2nd cycle).	AIHA & PRCA<br>↓Hb<br>↑LDH<br>↓haptoglobin<br>- DAT: (+) for "broadly specific warm auto-Abs"<br>- WBCs/PLTs: Normal<br>↑Ferritin<br>- Reticulocytopenia<br>- Bone marrow & aspirate: CD8^{+} T cell- mediated destruction of red cell precursors	- ICI discontinued: Yes<br>- 2 units PRBCs, MPL 1 g/Q12 hrs ×2 days, then Pred 1 mg/kg/PO QD<br>- Mycophenolate mofetil 1 g BID<br>- 2 units PRBCs, mycophenolate mofetil 1.5 g BID<br>- Corticosteroids IV<br>- Cyclosporine- A 5 mg/kg QD, Pred 1 mg/kg	Response	No	N/A	No
Hwang *et al.* 2020*	67 y/o Male	Melanoma	- Episode 1: Pembro + indoximod<br><br>- Episode 2: Ipi & Nivo<br><br>- Episode 3: Carbo/Pacli/Pembro	12<br><br>1<br><br>3	- NR	wAIHA<br>↓Hb<br>↑LDH<br>↓Haptoglobin<br>- DAT: IgG+, C3–	- ICI discontinued: Yes<br>- Pred 1 mg/kg PO QD<br><br>- Pred 1 mg/kg PO QD, IVIG 1 g/kg (×2), rituximab 375 mg/m^{2} (×4)<br><br>- Pred 1 mg/kg PO QD, IVIG 1 g/kg (×2), rituximab 375 mg/m^{2} (×4), IV MPL 1 g/day	Response<br><br>Response<br><br>Response	Yes<br><br>Yes<br><br>No	Yes<br><br>Yes<br><br>N/A	No
Atiq *et al.* 2020	58 y/o Female	LUAD	Pembro/Peme maintenance ×1<br>1st: Pembro/Carbo/Peme ×4	5	- High WBCs, BILI, & ALP prior to treatment<br>- Melena<br>- Mallory-Weiss tear	Secondary CAD<br>- Cold agglutinins: (1/128-512)<br>↑Retic count	- ICI discontinued: Yes<br>- RBC transfusions	Response	No	N/A	No

					- Chronic active gastritis/peptic ulcer disease.<br>- Previous transfusions	↓Haptoglobin (32 mg/dL)<br>Blood smear: agglutination, polychromasia<br>- DAT: IgG+, C3+<br>- Type II cryoglobulins: (+)<br>↑Serum IgM<br>- SPEP: Normal<br>- ANAs: (–)<br>- PNH panel: Normal<br>- Hepatic profile: Normal<br>- Mycoplasma: IgM–, IgG+<br>- Bone marrow: Mildly hypocellular	- Steroids with tapering + rituximab 375 mg/m^2/week IV ×4				
Jain *et al.* 2020	47 y/o Female	NSCLC	1st: Pembro	2	Prior to ICI<br>- Severe anemia<br>- DAT: IgG–, C3+<br>- Cold agglutinins (+, = 64)<br>- CAS secondary to malignancy	CAS<br>↓Hb<br>↑LDH<br>↑BILI<br>↓Haptoglobin<br>- DAT: Not performed<br>↑PLTs	- ICI discontinued: Yes<br>Multiple RBC transfusions<br>- Pred 1 mg/kg QD	Response	No	N/A	No
Baek *et al.* 2021	70 y/o Male	LUAD	1st: Pembro 200 mg/Cis/Peme	1	- No h/o other diseases	AIHA<br>↓Hb<br>↑BILI<br>↓Haptoglobin<br>↑LDH<br>↑Retic count<br>- Blood smear: spherocytes<br>- DAT: (+)<br>- Cold agglutinin: (+)<br>↓PLTs	- ICI discontinued: Yes<br>PSL 1 mg/kg<br>- PSL 2 mg/kg, plasma exchange (×5)	Response	No	N/A	No
Acikgoz *et al.* 2021	50 y/o Male	SCLC	1st: Atezo + Carbo/etoposide	3	- "Did not have any disease or regular medication"	CAD<br>- Cold agglutinins (+)<br>↓ Hb 9.6 g/dl<br>↓hematocrit 14.8%<br>- Blood smear: "Agglutinins"<br>- DAT: IgG–, C3d+<br>- IAT (+)<br>- Cryoglobulin: (–)<br>- Mycoplasma: IgM–<br>- Hep-B: Ag(–), Ab(–)<br>- Hep-C: Ab(–)<br>- HIV-1/2: Ab (–), p24 Ag (–)	- ICI discontinued: Yes<br>MPL 1 mg/kg IV QD ×10 days	CR	No	N/A	No
Karki *et al.* 2021	65 y/o Male	LUAD	1st: Pembro + Carbo/Peme	1	- Stents & bypass due to coronary artery disease<br>- h/o asymptomatic mild elevation of indirect BILI<br>- MZL	Secondary CAD← AIHA(DAT–) ← Gilbert syndrome<br>Cold agglutinin: >1:512.<br>↓Hb<br>↓Haptoglobin	- ICI discontinued: Yes<br>- 3 units RBCs<br>- MPL, then Pred PO<br>- Rituximab 375 mg/m^2 (×4)	Response	Yes	No	No

						↑BILI<br>↑LDH<br>- Blood smear:<br>Cold agglutination<br>- DAT1: (–)<br>- DAT2: IgG–, C3d+<br>- Serum: ↑IgM, ↓IgG, ↓C3, ↓C4<br>- Infectious workup: (–)<br>- SPEP with SIFE IgMκ<br>- Bone marrow: Low- grade B- cell lymphoma<br>- Flow cytometry: κ light chain restriction within $CD19^+$/$CD20^+$ B- cells					
Smithy *et al.* 2021	NR	Melanoma	1st: Ipi	1	- CLL	wAIHA<br>- DAT: IgG+, C3–<br>↑WBCs	- ICI discontinued: Yes<br>Rituximab Q1W ×8, concomitant corticosteroid taper	Response	Yes	No	No
Younce *et al.* 2021	56 y/o Female	Breast cancer	4th: Atezo (840mg Q15 days)/nab- Pacli (100mg/m^2/Q7 days)<br>3rd: Eribulin/Pacli<br>2nd: Fulvestrant/Palbociclib<br>1st: Neoadjuvant **FEC**, followed by Dtx, Sx & RT (48.6Gy). Maintenance with tamoxifen/Exemestane	1	- Anemia<br>- B_{12} deficiency<br>- HTN<br>- HCL<br>- Restless leg syndrome, anxiety, & tinnitus<br>- Gastric bypass	AIHA<br>↑LDH<br>↑Retic count<br>↓Haptoglobin<br>- BILI: Normal<br>DAT: NR<br>↑Ferritin<br>- FOBT: (–)	- ICI discontinued: Yes<br>Pred taper ×16 days, then Pred decreased by 20 mg Q3D, B_9 1 mg QD ×5 days	Response	Yes	No	No
Jobson *et al.* 2021	73 y/o Male	Melanoma	1st: Ipi (3mg/kg)<br>- RT (36 Gy in 6 fractions, 2 per week)	1	- CLL 2 yrs prior to melanoma<br>- Acoustic neuroma *in-situ*.<br>- Gluten intolerance<br>- Atrial fibrillation	AIHA<br>↓Hb<br>↑BILI<br>↑LDH<br>- DAT: (+)	- ICI discontinued: Yes<br>MPL 250 mg/Q8 hrs IV, B_9 5 mg QD, blood transfusions (×4)<br>- IVIG 1g/kg QD ×2 days<br>- CTX 750 mg IV ×1<br>- Splenectomy	No Response	Yes	No	No
Yun *et al.* 2021	86 y/o Male	Urothelial carcinoma	For UC:<br>3rd: Pembro<br>2nd: Intravesical GEM<br>1st: Intravesical BCG<br><br>For CLL<br>- Ibrutinib 420 mg PO QD	3	- CLL 5 yrs prior to UC	AIHA (DAT–)<br>↓Hb<br>↓Hematocrit<br>↓Haptoglobin<br>↑Total BILI 1.7 mg/dL<br>- LDH: 250 U/L<br>- Retic count: 0.05 × 10^3/μL (1.97%)<br>Blood smear:<br>a few schistocytes<br>- DAT: (–)<br>- WBCs/PLTs: Normal<br>↓Neutrophils<br>- MCV: Normal	- ICI discontinued: Yes<br>PRBCs<br>- Pred 1 mg/kg QD tapered over 5 weeks<br>- PRBCs, Pred 1 mg/kg QD<br>- Rituximab<br>- Ibrutinib 420 mg PO QD	Response	No	N/A	No

						- Bone marrow: Hypercellular (70%), 40% CLL involvement					
Sintawichai *et al.* 2021	50 y/o Female	Breast cancer (triple negative)	2nd: Atezo + Pacli<br>- Left simple mastectomy, RT after palliative Sx<br><br>1st: Left breast- conserving therapy with axillary lymph node dissection & CTX/MTX, postoperative RT after adjuvant chemotherapy in 1999	3	- No irAEs, neurological or hematological toxicities during the first 2 cycles<br>- No concomitant use of AIHA- associated drugs	AIHA<br>↓Hb<br>↑LDH<br>- Haptoglobin: Normal<br>↑Retic (%)<br>- Blood smear: polychromasia (2+), spherocytes (2+) of varying size, no schistocytes, normochromic- normocytic anemia<br>- DAT: (–)<br>- IAT: (–)<br>↓Hematocrit<br>↑RDW<br>↓WBCs<br>- PLTs: Normal<br>- No bleeding<br>↑Serum ferritin 1,191<br>- Serum iron: Within normal range<br>↓TIBC	- ICI discontinued: Yes<br>- Transfusion of leucocyte poor packed red cell + PSL 60 mg QD ×2 weeks, then PSL 10 mg	Response	No	N/A	No
Saliba *et al.* 2021	70 y/o Male	Esophageal adenocarcinoma	- Pembro 200 mg Q21 days + Carbo/Pacli Q1W<br>- Previous treatment: NR	3	- Other irAEs: None	AIHA (DAT–)<br>↓Hb<br>↑LDH<br>↑BILI<br>↓Haptoglobin<br>↑Retic count<br>↓PLTs<br>- DAT: (–)	- ICI discontinued: Yes<br>- Pred 1 mg/kg QD ×8 days<br>- IVIG 0.4 g/kg QD ×4 days	No Response	No	N/A	No
Saliba *et al.* 2021	66 y/o Female	Pancreatic adenocarcinoma	- Pembro 200 mg Q21 days + GEM & defactinib<br>- Previous treatment: NR	13	- Other irAEs: Pneumonitis	AIHA (DAT–)<br>↓Hb<br>↑LDH<br>↑BILI<br>↓Haptoglobin<br>↑Retic count<br>- DAT: (–)	- ICI discontinued: Yes<br>- Pred 0.8 mg/kg QD with 12-week taper	Response	No	N/A	No
Saliba *et al.* 2021	74 y/o Male	Melanoma	- Pembro 200 mg Q21 days<br>- Previous treatment: NR	15	- CLL<br>- Other irAEs: Thyroiditis	wAIHA<br>↓Hb<br>↑LDH<br>↑BILI<br>↓Haptoglobin<br>Retic count: 81<br>- DAT: IgG+<br>↓PLTs<br>- Bone marrow: 30–40% involvement with CLL	- ICI discontinued: No<br>- Pred 0.6 mg/kg QD with 12-week taper	CR	N/A	No	No

Saliba *et al.* 2021*	68 y/o Male	Melanoma	- Pembro 200 mg Q21 days + indoximod<br>- Previous treatment: NR	12	- Other irAEs: None	wAIHA<br>↓Hb<br>↑LDH<br>↑BILI<br>↓Haptoglobin<br>↑Retic count<br>- DAT: IgG+	- ICI discontinued: Yes<br>- Pred 1 mg/kg QD with 4-week taper	CR<br>CR<br>Response	Yes<br>Yes	Yes<br>Yes	No
Saliba *et al.* 2021	64 y/o Female	LUAD	- Pembro 200 mg Q21 days<br>- Previous treatment: NR	5	- h/o wAIHA<br>- Other irAEs: None	wAIHA<br>↓Hb<br>↑LDH<br>↑BILI<br>↓Haptoglobin<br>↑Retic count<br>- DAT: IgG+	- ICI discontinued: Yes<br>- Pred 1 mg/kg QD with 4-week taper	Response	Yes	Yes	No
Saliba *et al.* 2021	65 y/o Male	LUAD	- Pembro 200 mg Q21 days + Carbo/Peme<br>- Previous treatment: NR	3	- Other irAEs: None	AIHA<br>↓Hb<br>↑LDH<br>↑BILI<br>Retic count: 86<br>↓PLTs<br>- DAT: IgG+, C3+	- ICI discontinued: Yes<br>- Pred 0.6 mg/kg QD with 6-week taper	Response	Yes	Yes	No
Saliba *et al.* 2021	74 y/o Male	LUAD	- Pembro 200 mg Q21 days<br>- Previous treatment: NR	4	- Other irAEs: None	wAIHA<br>↓Hb<br>↑LDH<br>↑BILI<br>↓Haptoglobin<br>↑Retic count<br>- DAT: IgG+, C3+	- ICI discontinued: Yes<br>- Pred 0.6 mg/kg QD with 6-week taper	No Response	No	N/A	No
Saliba *et al.* 2021	56 y/o Male	LUSQ	- Pembro 200 mg Q21 days<br>- Previous treatment: NR	1	- Other irAEs: None	CAD<br>↓Hb<br>↑BILI<br>- DAT: C3+<br>- Cold agglutinin: >1:512<br>- No serum monoclonal protein detected<br>↓PLTs<br>- Bone marrow: hypocellular, erythroid hyperplasia, stress dyserythropoiesis	- ICI discontinued: Yes<br>- Pred 1 mg/kg/day with 4-week taper, IVIG 1 g/kg ×5 days<br>- Rituximab 375 mg/m^2 Q1W ×4	Response	No	N/A	No
Kramer *et al.* 2021	24 y/o Female	NR	- Ipi + Nivo	11 weeks	- Concurrent irAEs: Meningitis	AIHA<br>- NR	- ICI discontinued: Yes<br>- Corticosteroids, PRBCs, MMF (for hepatitis)	CR	No	N/A	No
Kramer *et al.* 2021	75 y/o Male	NR	- Ipi	3 weeks	- Concurrent irAEs: None	AIHA<br>- NR	- ICI discontinued: Yes<br>- MPL 1g, PRBC, IVIG, splenectomy, CTX	CR	Yes	No	No
Kramer *et al.* 2021	50 y/o Female	NR	- Ipi + Nivo	15 weeks	- Concurrent irAEs: Fever	AIHA<br>- NR	- ICI discontinued: Yes<br>- Corticosteroids, PRBCs	CR	No	N/A	No

Kramer *et al.* 2021	87 y/o Male	NR	- Nivo	8 weeks	- Concurrent irAEs: None	AIHA<br>- NR	- ICI discontinued: Yes<br>- Corticosteroids, PRBCs	Response	No	N/A	No
Kramer *et al.* 2021	67 y/o Male	NR	- Ipi + Pembro	3 weeks	- Concurrent irAEs: None	AIHA<br>- NR	- ICI discontinued: Yes<br>- Corticosteroids, PRBCs, rituximab, IVIG	Response	Yes	Yes	No
Kramer *et al.* 2021	69 y/o Female	NR	- Pembro	32 weeks	- Concurrent irAEs: Carpal Tunnel Syndrome	AIHA<br>- NR	- ICI discontinued: Yes<br>- Corticosteroids, PRBCs	CR	No	N/A	No
Kramer *et al.* 2021	76 y/o Male	NR	- Nivo	2 weeks	- Concurrent irAEs: None	AIHA<br>- NR	- ICI discontinued: Yes<br>- Corticosteroids, alemtuzumab	CR	No	N/A	No
Kramer *et al.* 2021	70 y/o Male	NR	- Nivo	3 weeks	- Concurrent irAEs: None	AIHA<br>- NR	- ICI discontinued: No<br>- PRBCs	Response	N/A	Yes	No
Carbo-Bague *et al.* 2021	62 y/o Male	Melanoma (Acral)	1st: Nivo 3 mg/kg Q2W (adjuvant) after Sx (excision)	3	- No concomitant AIHA-associated drugs	AIHA<br>↓Hb<br>↑Indirect BILI<br>↑LDH<br>↓Haptoglobin<br>- DAT: C3d+, IgG–, IgM–<br>↓Neutrophils	- ICI discontinued: Yes<br>- 3 RBC transfusions<br>- MPL 1 mg/kg with taper	CR	Yes	No	No
Endo *et al.* 2022	65 y/o Female	LUAD	1st: Atezo + Carbo + nab- Pacli	1	- ANAs+ & anti-DNA+ prior to treatment	AIHA & HLH<br>↓Hb<br>↓Haptoglobin<br>- DAT: IgG+, C3d+<br>↓PLTs<br>↓Neutrophils<br>- Bone marrow: hemophagocytosis<br>↓Fibrinogen<br>↑Triglyceride<br>↑Ferritin<br>↑AAT<br>↑ Soluble IL-R2<br>- EBV: (–)<br>-CMV: (–)	- ICI discontinued: Yes<br>PSL 1 mg/kg QD<br>- G- CSF & antibiotics<br>- 2 units of RBC concentrate<br>- Transfusion of PLT concentrate	CR	No	N/A	No
Chambers *et al.* 2022	73 y/o Male	NSCLC	2nd: Atezo<br>1st: Carbo/Peme, SRS for brain metastases	30	- Levothyroxine for immune- induced HT.	AIHA<br>↓Hb<br>↑BILI<br>↑LDH<br>↓haptoglobin<br>↑Reticulocytes<br>- Blood smear: Polychromasia & spherocytes, no fragments or agglutination.<br>- DAT: IgG+, C3d–<br>- WBCs: Normal<br>- PLTs: Normal<br>- ALT: Normal	- ICI discontinued: Yes<br>RBCs transfusion<br>- PSL 1 mg/kg PO QD with 3- month taper (15 mg QD)	CR	No	N/A	No

						- ANAs: (–)					
Tao *et al.* 2022	60 y/o Female	Hodgkin Lymphoma	3rd: Penpulimab (anti-PD-1) 200 mg Q2W<br>2nd: GVD (3 cycles)<br>1st: ABVD chemotherapy & 4 cycles RT 30 Gy	2	- "With no previous medical history"	AIHA<br>↓Hb<br>↑Indirect BILI<br>↑LDH<br>↓haptoglobin<br>- DAT: (+)<br>↓RBCs<br>↓PLTs<br>↑ESR<br>↑ALT	- ICI discontinued: Yes<br>- Pred 2 mg/kg with taper<br>- Washed RBCs & plasmapheresis	CR	No	N/A	No
Kubo *et al.* 2022	56 y/o Male	Melanoma	2nd: SRT, dabrafenib + trametinib ×1 month<br><br>- Relapse & brain metastases<br><br>1st: Adjuvant Nivo 240 mg Q2W (7 cycles) after wide local excision	7	- NR	AIHA & reactive lymphadenopathy (1 month after ICI cessation)<br>↓Hb<br>↓Hematocrit<br>↓RBCs<br>↑Reticulocytes<br>↑Eosinophils<br>- Platelets: Normal<br>↑Total & direct BILI<br>↑LDH<br>↓Haptoglobin<br>- DAT: (+)<br>- Cold agglutinin: 1:128 (+)<br>- ANAs: (–)<br>↑Ferritin<br>↑ CRP<br>↑D- dimer<br>↑AST<br>↑ALT<br>↑ALP<br>↑GGT<br>↑ sIL2R<br>- EBV: nuclear (+), capsid (IgG+)<br>- CMV: IgG+, IgM–<br>- HIV: (–)<br>- Ultrasound: enlarged spleen<br>- PET- CT: systemic lymphadenopathy<br>- Lymph node biopsy: No malignancy, reactive lymphoid hyperplasia<br>Bone marrow: cellular hyperplasia, erythroid predominance, no abnormal cells.	- ICI discontinued: Yes<br>Transfusion<br>- Pred 1 mg/kg QD	CR	No	N/A	No
Kakita *et al.* 2023	73 y/o Male	Urothelial carcinoma	2nd: Pembro<br>1st: GEM/Carbo	33	- NR	Evans syndrome<br>↓Hb	- ICI discontinued: Yes	CR	No	N/A	No

			- Radical nephroureterectomy			↓RBCs<br>↓PLTs<br>↑LDH<br>↑Indirect BILI<br>- DAT: (+)<br>- IAT: (+)<br>↑Liver enzymes<br>- Hepatosplenomegaly<br>- Mesentery & hepatic hilum with enlarged lymph nodes<br>- No bleeding or infection<br>Bone marrow: Normal	RBC & PLT transfusions<br>- PSL 35 mg PO QD ×34<br>- Pred 5 mg PO QD				
Zhang *et al.* 2023	71 y/o Male	LUAD	1st: Pembro 200 mg	1	- h/o complement mediated AIHA<br><br>Concomitant irAEs:<br>- DVT<br>- Diarrhea<br>- Myocarditis<br>- Acute kidney injury	Evans syndrome<br>↓Hb<br>↑LDH<br>↑Retic count<br>↑BILI total & indirect<br>- DAT: IgG+, C3+<br>PLT IgG: (+)<br>↓PLTs<br>↑cTnI<br>↑CK- MB<br>↑BUN<br>↑D- dimer<br>↑CRP<br>Cold agglutinin: (–)<br>- Blood smear: Schistocytes (0.7%) & spherocytes (0.4%)	- ICI discontinued: Yes<br>MPL 2 mg/kg QD ×3 then Pred 60 mg PO	Response	No	N/A	No
Dirven *et al.* 2023	67 y/o Male	Melanoma	For Melanoma:<br>4th: Nivo 480 mg Q4W in 2022<br>- Sx & RT for abdominal & brain metastases<br>3rd: Trametinib 1 mg + dabrafenib 50 mg BID (2018)<br>2nd: Resection & Pembro 2 mg/kg Q3W<br>1st: Sx (resection) with adjuvant Pembro 2 mg/kg Q3W (×4)<br><br>For CLL:<br>1st: Ibrutinib with complete response in 2020	1	- CLL in 2015<br>- Membranous glomerulonephritis<br>- Atrial fibrillation<br><br>Previous irAEs:<br>- Grade 1 skin toxicity with Pembro	wAIHA<br>↓Hb<br>↑LDH<br>↑Total & indirect BILI<br>↓Haptoglobin<br>- Reticulocytosis<br>- Blood smear: No schistocytes<br>- DAT: (IgG+, weak for complement)<br>- Coagulation tests: Normal	- ICI discontinued: Yes<br>MPL 1 mg/kg with 2-month taper + rituximab Q1W (×4)	CR	No	N/A	No
Fukushima *et al.* 2023	86 y/o Male	HCC	1st: Atezo + Beva<br>- Central bisectionectomy	3	- DBM type NR<br>- h/o heavy drinking, possible etiology of HCC<br>- No h/o autoimmune disease<br>- No drugs associated with thrombocytopenia	Evans syndrome ← wAIHA ← ITP<br>ITP (60 days post- ICI):<br>↓PLTs<br>- PLTs IgG: elevated<br>- Coagulation: normal<br>- Hb: Normal<br>- Haptoglobin: Normal	- ICI discontinued: Yes<br>For ITP:<br>- PLT transfusion & high-dose IVIG<br>- H. pylori eradication therapies<br><br>For wAIHA:	Response	No	N/A	No

						- Blood smear: No schistocytes<br>- Plasma IgG: 2440 mg/mL<br>- ANAs: × 640 (homogeneous)<br>- Bone marrow: No dysplasia or cancer invasion<br><br>AIHA (80 days post- ICI):<br>↓Hb<br>↑Total & direct BILI<br>↑LDH<br>↓Haptoglobin<br>↑Retic (%)<br>- DAT: IgG+<br>- IAT: IgG+ (warm)	- Erythrocyte transfusion<br>- PSL (no initial dose or duration given), then PSL 5 mg (no duration given)				
Ubaldi *et al.* 2023	35 y/o Male	Melanoma	3rd: Neoadjuvant Ipi & Nivo<br>2nd: Sx (resection) & adjuvant Pembro<br>1st: Sx (resection) & adjuvant dabrafenib<br>+ trametinib (1 yr)	4	- No previous irAEs<br>- No use of AIHA-associated drugs	cAIHA (delayed)<br>27 days after ICI termination<br>↓Hb<br>↓Haptoglobin<br>↓ RBCs<br>↑ Retic (%)<br>↑LDH<br>↑Total BILI<br>- Direct BILI: Normal<br>- DAT: C3d+<br>- Cold agglutinins: IgM+<br>- anti-PLT Abs: (–)	- ICI discontinued: Yes<br>MPL 1 mg/kg IV + rituximab 660 mg IV Q1W (×3)	CR	No	N/A	No
Nakatsuru *et al.* 2023	67 y/o Female	LUAD	- Maintenance: Atezo + Beva<br><br>1st: Atezo + Carbo/<br>Peme + Beva (4 cycles)	7	Other irAES<br>- Grade 2 neutropenia after 4 cycles of 1- line therapy<br>- Grade 3 neutropenia after 2 cycles of maintenance, therapy-related MDS	AIHA<br>↓Hb<br>↑Total BILI<br>↑LDH<br>- DAT: (+)<br>↓PLTs<br>- anti-PLT Abs: (–)	- ICI discontinued: Yes<br>PSL 50 mg QD with 4-month taper	CR	No	N/A	No
Patodiya *et al.* 2023	25 y/o Female	Hodgkin Lymphoma	1st: Nivo (3 mg/kg) Q2W	2	- Recent ciprofloxacin antibiotic course<br>- Cholestatic jaundice attributed to ciprofloxacin<br>-Vanishing bile duct syndrome	Secondary AIHA<br>↓Hb<br>↑BILI (total, direct & indirect)<br>↑LDH<br>↓Haptoglobin<br>- DAT: (+)<br>- IAT: (–)	- ICI discontinued: Yes<br>Multiple units of irradiated PRBCs<br>- No corticosteroid therapy	No Response	No	N/A	No
Khosla *et al.* 2024	50 y/o Female	SCLC	1st: Atezo + Carbo, etoposide	3	- Blood transfusions with each chemotherapy cycle<br>- No medical or family h/o membranopathy or hemoglobinopathy<br>- Negative h/o HTN	AIHA (DAT–)<br>↓Hb<br>↑Total BILI<br>↑LDH<br>↓Haptoglobin<br>- Retic (%): Normal	- ICI discontinued: Yes<br>Transfusion PRBCs (8 units)<br>- Pred 1 mg/kg with taper	CR	Yes	No	No

						Blood smear:<br>multiple spherocytes, reticulocytes, & few nucleated RBCs<br>- DAT: (–)<br>↑RDW<br>↑MCV: 105 fL<br>↓PLTs<br>- WBCs: Normal<br>- CT: No bleeding<br>- Infectious panel: (–)<br>- Coagulation panel: "Unremarkable"<br>- ANAs: (–)<br>- No hypersplenism					
Fetter *et al.* 2024	64 y/o Female	Melanoma	- Sx (excision)<br>- 4 cycles Ipi (3 mg/kg) + Nivo (1 mg/kg) Q3W<br>- 8 cycles Nivo (480 mg) Q4W<br>- 3 cycles Ipi (3 mg/kg) + Nivo (1 mg/kg) Q3W<br><br>After wAIHA treatment<br>- RT<br>-Pembro (200 mg Q3W) + lenvatinib (20 mg QD)	15	- Bronchial asthma<br>Other irAES<br>- Thromocytopenia after ICI rechallenge	wAIHA<br>↓Hb<br>↑LDH<br>- Haptoglobin: Normal<br>- BILI: Normal<br>- DAT: IgG+, C3d+<br>After treatment<br>↓ Retic: 5.500 cells/uL<br>- Mone marrow aspiration: Normal<br>- Karyogram: Negative<br>After ICI rechallenge<br>Evans Syndrome<br>↓Hb<br>↑LDH<br>↓ Retic: 10.400 cells/uL<br>↓Thrombocytes (94.000 cells/uL)	- ICI discontinued: Yes<br>- PSL 80 mg with taper<br>After rechallenge<br>- PSL 70 mg IV<br>- 2 RBC concentrates	CR	Yes	Yes	No
Fetter *et al.* 2024	75 y/o Male	Melanoma	- Sx (excision)<br>- RT<br>- IFN-a (Roferon 3 million IU subcutaneous 3x/week)<br>- Adjuvant Pembro (400 mg Q6W) + RT	1	- Melanoma in situ of the right cheek<br>- Cardiac pacemaker (with disconnected right ventricular probe)<br>- Arterial HTN<br>- Hyperuricemia<br>- Transient ischemic attack<br>- Partial bowel resection due to intestinal stenosis<br>Other irAES<br>-Myocarditis<br>Medications<br>- lisinopril (20 mg) + bisoprolol (2.5 mg) QD	ICPi-mediated AIHA & ICPi-mediated myocarditis<br>↓Hb<br>↑Free Hb<br>↓Haptoglobin<br>↑LDH (925 U/l)<br>↑Indirect BILI (0.82 mg/dL)<br>↑Retic count (106.600 cells/uL)<br>- DAT: (–)<br>↑NT-pro-BNP (912 pg/mL)<br>↑Troponin T (110 ng/mL)<br>- ECG: negative for acute ischemia<br>- TTE: severely dilated left & right atria, moderate mitral regurgitation & severe tricuspid regurgitation	- ICI discontinued: Yes<br>-PSL IV (90 mg) ×10 days, then PSL PO with tapered	CR	No	N/A	No

						- EGD: Normal<br>- CT scans: Normal<br>WBP: Normal<br>- Other blood tests: Negative for acute leukemia, non-Hodgkin lymphoma, EBV, CMV, parvovirus B19, human immunodeficiency viruses, hepatitis A, B & C viruses					

Abbreviations: **3D-CRT**, three-dimensional conformal radiotherapy; **AAT**, aspartate aminotransferase; **Ab**, antibody; **ABVD**, adriamycin, bleomycin, vinblastine, dacarbazine; **AIHA**, autoimmune hemolytic anemia; **ALP**, alkaline phosphatase; **ALT**, alanine aminotransferase; **AML**, acute myeloid leukemia; **ANA**, anti-nuclear antibody; **Atezo**, atezolizumab; **B_9**, folate; **B_{12}**, cobalamin; **BCG**, Bacillus Calmette-Guérin; **Beva**, bevacizumab; **BID**, twice a day; **BILI**, bilirubin; **BUN**, blood urea nitrogen; **CAD**, cold agglutinin disease; **Carbo**, carboplatin; **CAS**, cold agglutinin syndrome; **CCL**, chronic lymphocytic leukemia; **Cis**, cisplatin; **CMV**, cytomegalovirus; **CTX**, cyclophosphamide; **COPD**, chronic obstructive pulmonary disease; **CR**, complete response; **CRP**, C-reactive protein; **CSF**, cerebrospinal fluid; **CT**, computed tomography; **DAT**, direct antiglobulin test; **DMT1**, diabetes mellitus type 1; **DMT2**, diabetes mellitus type 2, **Dtx**, docetaxel; **DVT**, deep venous thrombosis; **EBV**, Epstein-Barr Virus; **ECG**, electrocardiogram; **EGD**, esophagogastroduodenoscopy; **ESR**, erythrocyte sedimentation rate; **FEC**, fluorouracil, epirubicin & cyclophosphamide; **FLAG-IDA**, fludarabine, cytarabine, granulocyte colony-stimulating factor, and idarubicin; **FOBT**, fecal occult blood test; **G-CSF**, granulocyte colony-stimulating factor; **GEM**, gemcitabine; **GGT**, gamma-glutamyl Transferase, **GM-CSF**, granulocyte-macrophage colony stimulating factor; **GVD**, gemcitabine, vinorelbine, liposomal doxorubicin; **h/o**, history of; **HA**, hemolytic anemia; **Hb**, hemoglobin; **HCC**, hepatocellular carcinoma; **HCL**, hypercholesterolemia; **HLD**, hyperlipidemia; **HLH**, hemophagocytic lymphohistiocytosis; **HT**, hypothyroidism; **HTN**, hypertension; **IAT**, indirect antiglobulin test; **IFN**, interferon; **Ipi**, ipilimumab; **irAE**, immune-related adverse event; **ITP**, immune thrombocytopenic purpura; **IVIG**, intravenous immune globulin; **LDH**, lactate dehydrogenase; **LFT**, liver function tests; **LUAD**, lung adenocarcinoma; **LUSC**, lung squamous cell carcinoma; **MDS**, myelodysplastic syndrome; **MMF**, mycophenolate mofetil; **MPL**, methylprednisolone; MTX, methotrexate; **MZL,** marginal Zone Lymphoma; **N/A**, not applicable; **Nivo**, nivolumab; **NR**, not reported; **NSAIDs**, non-steroidal anti-inflammatory drugs; **NSCLC**, non-small-cell lung cancer; **NT-pro**, N-terminal pro B-type natriuretic peptide; **Pacli**, paclitaxel; **Pembro**, pembrolizumab; **Peme**, pemetrexed; **PLTs**: platelets **PNH**, paroxysmal nocturnal hemoglobinuria; **PO**, by mouth; **PRCA**, pure red-cell aplasia; **Pred**, prednisone; **PSL**, prednisolone; **QD**, once per day; **Q1W**, once per week; **Q2W**, every two weeks; **Q3W**, every three weeks; **Q4W**, every four weeks; **RBC**, red blood cell; **RCC**, renal cell carcinoma; **Retic**, reticulocyte; **RT**, radiotherapy; **SCC**, squamous cell carcinoma; **SCLC**, small-cell lung cancer; **sIL2R**, soluble interleukin-2 receptor; **SPEP**, serum protein electrophoresis; **SRS**, stereotactic radiosurgery; **SRT**, stereotactic radiotherapy; **Sx**, surgery; **T4**, thyroxine; **TFTs**, thyroid function tests; **TTE**, Transthoracic echocardiography; **TURP**, transurethral resection of the prostate; **UPEP**, urine protein electrophoresis; **WBCs**, white blood cell; **WBP**, whole Body Plethysmography; **WBRT**, whole-brain radiation therapy; **y/o**, years old.

↓ Downwards arrow indicates levels below lower limit of normal.

↑ Upwards arrow indicates levels above upper limit of normal.

*Patient 4 in Leaf *et al*. (2019) likely corresponds to the patient in the case-report by Hwang *et al*. (2020) and to patient 4 in the case-series by Saliba *et al*. (2021).

Supplementary References

1. Simeone, E., Grimaldi, A.M., *et al* (2014) Serious haematological toxicity during and after ipilimumab treatment: a case series. *J Med Case Rep*, 8, 240.

2. Lott, A., Butler, M., *et al* (2015) Evan's Syndrome Associated with Pembrolizumab Therapy in Metastatic Non-Small Cell Lung Cancer. *Blood*, 126, 4543–4543.

3. Kong, B.Y., Micklethwaite, K.P., *et al* (2016) Autoimmune hemolytic anemia induced by anti-PD-1 therapy in metastatic melanoma. *Melanoma Res*, 26, 202–204.

4. Nair, R., Gheith, S., *et al* (2016) Immunotherapy-Associated Hemolytic Anemia with Pure Red-Cell Aplasia. *The New England journal of medicine*, 374, 1096–1097.

5. Palla, A.R., Kennedy, D., *et al* (2016) Autoimmune Hemolytic Anemia as a Complication of Nivolumab Therapy. *Case Rep Oncol*, 9, 691–697.

6. Schwab, K.S., Heine, A., *et al* (2016) Development of Hemolytic Anemia in a Nivolumab-Treated Patient with Refractory Metastatic Squamous Cell Skin Cancer and Chronic Lymphatic Leukemia. *Case Rep Oncol*, 9, 373–378.

7. Khan, U., Ali, F., *et al* (2017) Immunotherapy-associated autoimmune hemolytic anemia. *J Immunother Cancer*, 5, 15.

8. Ramos, B., Gastal, G., *et al* (2017) An Autoimmune Haemolytic Anaemia Secondary to Ipilimumab Treatment. *Klin Onkol*, 30, 128–130.

9. Tardy, M.P., Gastaud, L., *et al* (2017) Autoimmune hemolytic anemia after nivolumab treatment in Hodgkin lymphoma responsive to immunosuppressive treatment. A case report. *Hematol. Oncol.*, 35, 875–877.

10. Le Burel, S., Champiat, S., *et al* (2017) Prevalence of immune-related systemic adverse events in patients treated with anti-Programmed cell Death 1/anti-Programmed cell Death-Ligand 1 agents: A single-centre pharmacovigilance database analysis. *European journal of cancer*, 82, 34–44.

11. Deltombe, C., Garandeau, C., *et al* (2017) Severe Allograft Rejection and Autoimmune Hemolytic Anemia After Anti-PD1 Therapy in a Kidney Transplanted Patient. *Transplantation*, 101, e291.

12. Algaze, S.D., Park, W., *et al* (2018) Autoimmune haemolytic anaemia in a patient with advanced lung adenocarcinoma and chronic lymphocytic leukaemia receiving nivolumab and intravenous immunoglobulin. *BMJ Case Rep*, 2018.

13. Ogawa, K., Ito, J., *et al* (2018) Exacerbation of autoimmune hemolytic anemia induced by the first dose of programmed death-1 inhibitor pembrolizumab: a case report. *Investigational new drugs*, 36, 509–512.

14. Shaikh, H., Daboul, N., *et al* (2018) A case of autoimmune haemolytic anaemia after 39 cycles of nivolumab. *BMJ Case Rep*, 2018.

15. Sun, Y., Lee, S.K., *et al* (2018) Management of Immune-mediated Cytopenias in the Era of Cancer Immunotherapy: A Report of 4 Cases. *J Immunother*, 41, 32–34.

16. Robilliard, B., Arnaud, E., *et al* (2018) A case of pembrolizumab-induced autoimmune haemolytic anaemia with polymyalgia rheumatica. *European journal of cancer*, 103, 281–283.

17. Ghosn, J., Vicino, A., *et al* (2018) A severe case of neuro-Sjogren's syndrome induced by pembrolizumab. *J Immunother Cancer*, 6, 110.

18. Hasanov, M., Konoplev, S.N., *et al* (2018) Nivolumab-induced cold agglutinin syndrome successfully treated with rituximab. *Blood Adv.*, 2, 1865–1868.

19. Johnstone, P. & Khan, O. (2019) Pembrolizumab-associated autoimmune haemolytic anaemia. *BMJ Case Rep*, 12.

20. Delanoy, N., Michot, J.M., *et al* (2019) Haematological immune-related adverse events induced by anti-PD-1 or anti-PD-L1 immunotherapy: a descriptive observational study. *Lancet Haematol*, 6, e48–e57.

21. Ni, D., AlZahrani, F., *et al* (2019) AIHA and Pancytopenia as Complications of Pembrolizumab Therapy for Metastatic Melanoma: A Case Report. *Case Rep Oncol*, 12, 456–465.

22. Tanios, G.E., Doley, P.B., *et al* (2019) Autoimmune hemolytic anemia associated with the use of immune checkpoint inhibitors for cancer: 68 cases from the Food and Drug Administration database and review. *Eur J Haematol*, 102, 157–162.

23. Leaf, R.K., Ferreri, C., *et al* (2019) Clinical and laboratory features of autoimmune hemolytic anemia associated with immune checkpoint inhibitors. *Am J Hematol*, 94, 563–574.

24. Williams, H. & Aitchison, R. (2019) Pembrolizumab-induced autoimmune haemolytic anaemia and cholangitis. *BMJ Case Rep*, 12.

25. Okawa, S., Kayatani, H., *et al* (2019) Pembrolizumab-induced Autoimmune Hemolytic Anemia and Hemophagocytic Lymphohistiocytosis in Non-small Cell Lung Cancer. *Intern Med*, 58, 699–702.

26. Dutertre, M., de Menthon, M., *et al* (2019) Cold agglutinin disease as a new immune-related adverse event associated with anti-PD-L1s and its treatment with rituximab. *European journal of cancer*, 110, 21–23.

27. Olson, D.J., Rajagopal, P., *et al* (2020) A case of dual-mechanism immune-related anaemia in a patient with metastatic melanoma treated with nivolumab and ipilimumab. *J Immunother Cancer*, 8.

28. Hwang, S.R., O'Dowd, T., *et al* (2020) Recurrent checkpoint inhibitor-induced warm agglutinin autoimmune hemolytic anemia in a patient with metastatic melanoma. *Am J Hematol*, 95, E169–E171.

29. Atiq, O., Atiq, S.O., *et al* (2020) Pembrolizumab-Induced Cold Agglutinin Disease. *Am J Case Rep*, 21, e924283.

30. Jain, N.A., Zhao, S., *et al* (2020) Association Between RBC Antigen Allo-Antibodies and Immune-Related Adverse Events During Immune Checkpoint Inhibitor Treatment for Advanced Cancers. *Cancer Manag Res*, 12, 11743–11749.

31. Baek, D.W. & Chae, Y.S. (2021) Pembrolizumab-related autoimmune hemolytic anemia in a patient with metastatic lung adenocarcinoma: a case report. *Yeungnam Univ J Med*, 38, 366–370.

32. Acikgoz, O., Bayramgil, A., *et al* (2021) Rare side effect caused by atezolizumab, an immune checkpoint inhibitor: Cold agglutinin disease. *J Oncol Pharm Pract*, 27, 2066–2068.
33. Karki, N.R., McElhone, P., *et al* (2021) Diagnosis and management of cold agglutinin disease associated with low-grade B-cell lymphoma in a patient receiving pembrolizumab for lung cancer. *BMJ Case Rep*, 14.
34. Smithy, J.W., Pianko, M.J., *et al* (2021) Checkpoint Blockade in Melanoma Patients With Underlying Chronic Lymphocytic Leukemia. *J Immunother*, 44, 9–15.
35. Younce, C.M., Lawton, J.M., *et al* (2021) Atezolizumab-induced hemolytic anemia - A case report. *J Oncol Pharm Pract*, 27, 1026–1028.
36. Jobson, D., McCormack, C.J., *et al* (2021) Severe treatment-resistant autoimmune haemolytic anaemia following ipilimumab in a patient with metastatic melanoma and CLL. *Leuk Lymphoma*, 62, 992–994.
37. Yun, N.K., Alrifai, T., *et al* (2021) Pembrolizumab-induced autoimmune haemolytic anemia in a patient with chronic lymphocytic leukaemia successfully treated with ibrutinib. *BMJ Case Rep*, 14.
38. Sintawichai, N., Lawasut, P., *et al* (2021) Autoimmune hemolytic anemia, rare immune-related adverse event after combination atezolizumab and paclitaxel treatment in recurrent triple-negative breast cancer: A case-report. *Journal of Medical Case Reports and Case Series*, 4, 1–5.
39. Saliba, A.N., Xie, Z., *et al* (2021) Immune-related hematologic adverse events in the context of immune checkpoint inhibitor therapy. *Am J Hematol*, 96, E362–E367.
40. Kramer, R., Zaremba, A., *et al* (2021) Hematological immune related adverse events after treatment with immune checkpoint inhibitors. *European journal of cancer*, 147, 170–181.
41. Carbo-Bague, A., Fort-Culillas, R., *et al* (2021) Nivolumab-Induced Autoimmune Haemolytic Anaemia and Safety of Subsequent Use of Ipilimumab: A Case Report. *Case Rep Oncol*, 14, 1289–1294.
42. Endo, Y., Inoue, Y., *et al* (2022) Marked, Lasting Disease Regression and Concomitantly Induced Autoimmune Hemolytic Anemia and Hemophagocytic Lymphohistiocytosis in a Patient With Lung Adenocarcinoma and Autoantibodies Receiving Atezolizumab Plus Chemotherapy: A Case Report. *JTO Clin Res Rep*, 3, 100263.
43. Chambers, B.S., Ward, D., *et al* (2022) Atezolizumab-induced autoimmune haemolytic anaemia caused by drug-independent antibodies. *European journal of cancer*, 162, 158–160.
44. Tao, Y., Han, J., *et al* (2022) Autoimmune hemolytic anemia in patients with relapsed Hodgkin's lymphoma after treatment with penpulimab, a monoclonal antibody against programmed death receptor-1. *Investigational new drugs*, 40, 854–857.
45. Kubo, T., Hino, A., *et al* (2022) Nivolumab-induced systemic lymphadenopathy occurring during treatment of malignant melanoma: a case report. *Int J Hematol*, 116, 302–306.
46. Kakita, S., Matsuo, T., *et al* (2023) Evans syndrome during pembrolizumab therapy for upper urinary tract cancer. *IJU Case Rep*, 6, 298–301.
47. Zhang, X., Gao, B.X., *et al* (2023) A 71-year-old male with a life-threatening recurrence of hemolytic anemia, thrombocytopenia, and acute kidney injury after pembrolizumab therapy: a case report. *BMC Geriatr*, 23, 478.
48. Dirven, I., Vander Mijnsbrugge, A.S., *et al* (2023) Auto-immune hemolytic anemia and hemophagocytic lymphohistiocytosis as immune-related adverse event in patients with metastatic melanoma and concurrent chronic lymphocytic leukemia: a case series and literature review. *Melanoma Res*, 33, 338–344.
49. Fukushima, M., Tajima, K., *et al* (2023) Evans' syndrome induced by atezolizumab plus bevacizumab combination therapy in advanced hepatocellular carcinoma. *Clin J Gastroenterol*, 16, 402–406.
50. Ubaldi, M., Sergi, M.C., *et al* (2023) Delayed cold-type autoimmune hemolytic anemia responding to Rituximab in a melanoma patient treated with Ipilimumab and Nivolumab. *European journal of cancer*, 193, 113320.
51. Nakatsuru, K., Tsubouchi, K., *et al* (2023) Acute myeloid leukemia and myelodysplastic syndrome associated with a combination of immune checkpoint inhibitor and platinum-based chemotherapy. *Thoracic cancer*, 14, 2225–2228.
52. Patodiya, B., Ramani, V.K., *et al* (2023) A rare presentation of vanishing bile duct syndrome in Hodgkin lymphoma: Case report. *SAGE Open Med Case Rep*, 11, 2050313X231208968.
53. Khosla, A., Sandhu, R.S., *et al* (2024) Atezolizumab-Induced Direct Antiglobulin Test-Negative Autoimmune Hemolytic Anemia. *American journal of therapeutics*, 31, e324–e328.
54. Fetter, T., Fietz, S., *et al* (2024) Severe autoimmune hemolytic anemia following immunotherapy with checkpoint inhibitors in two patients with metastatic melanoma: a case report. *Front Immunol*, 15, 1342845.
